# A pan-TE map highlights transposable elements underlying domestication and agronomic traits in Asian rice

**DOI:** 10.1093/nsr/nwae188

**Published:** 2024-06-04

**Authors:** Xiaoxia Li, Xiaofan Dai, Huiying He, Yang Lv, Longbo Yang, Wenchuang He, Congcong Liu, Hua Wei, Xiangpei Liu, Qiaoling Yuan, Xianmeng Wang, Tianyi Wang, Bintao Zhang, Hong Zhang, Wu Chen, Yue Leng, Xiaoman Yu, Hongge Qian, Bin Zhang, Mingliang Guo, Zhipeng Zhang, Chuanlin Shi, Qianqian Zhang, Yan Cui, Qiang Xu, Xinglan Cao, Dandan Chen, Yongfeng Zhou, Qian Qian, Lianguang Shang

**Affiliations:** Shenzhen Branch, Guangdong Laboratory of Lingnan Modern Agriculture, Genome Analysis Laboratory of the Ministry of Agriculture and Rural Affairs, Agricultural Genomics Institute at Shenzhen, Chinese Academy of Agricultural Sciences, Shenzhen 518120, China; Rice Research Institute, Shenyang Agricultural University, Shenyang 110866, China; Shenzhen Branch, Guangdong Laboratory of Lingnan Modern Agriculture, Genome Analysis Laboratory of the Ministry of Agriculture and Rural Affairs, Agricultural Genomics Institute at Shenzhen, Chinese Academy of Agricultural Sciences, Shenzhen 518120, China; Rice Research Institute, Shenyang Agricultural University, Shenyang 110866, China; Shenzhen Branch, Guangdong Laboratory of Lingnan Modern Agriculture, Genome Analysis Laboratory of the Ministry of Agriculture and Rural Affairs, Agricultural Genomics Institute at Shenzhen, Chinese Academy of Agricultural Sciences, Shenzhen 518120, China; Shenzhen Branch, Guangdong Laboratory of Lingnan Modern Agriculture, Genome Analysis Laboratory of the Ministry of Agriculture and Rural Affairs, Agricultural Genomics Institute at Shenzhen, Chinese Academy of Agricultural Sciences, Shenzhen 518120, China; Rice Research Institute, Shenyang Agricultural University, Shenyang 110866, China; Shenzhen Branch, Guangdong Laboratory of Lingnan Modern Agriculture, Genome Analysis Laboratory of the Ministry of Agriculture and Rural Affairs, Agricultural Genomics Institute at Shenzhen, Chinese Academy of Agricultural Sciences, Shenzhen 518120, China; Shenzhen Branch, Guangdong Laboratory of Lingnan Modern Agriculture, Genome Analysis Laboratory of the Ministry of Agriculture and Rural Affairs, Agricultural Genomics Institute at Shenzhen, Chinese Academy of Agricultural Sciences, Shenzhen 518120, China; Shenzhen Branch, Guangdong Laboratory of Lingnan Modern Agriculture, Genome Analysis Laboratory of the Ministry of Agriculture and Rural Affairs, Agricultural Genomics Institute at Shenzhen, Chinese Academy of Agricultural Sciences, Shenzhen 518120, China; Yazhouwan National Laboratory, Sanya 572024, China; Shenzhen Branch, Guangdong Laboratory of Lingnan Modern Agriculture, Genome Analysis Laboratory of the Ministry of Agriculture and Rural Affairs, Agricultural Genomics Institute at Shenzhen, Chinese Academy of Agricultural Sciences, Shenzhen 518120, China; Shenzhen Branch, Guangdong Laboratory of Lingnan Modern Agriculture, Genome Analysis Laboratory of the Ministry of Agriculture and Rural Affairs, Agricultural Genomics Institute at Shenzhen, Chinese Academy of Agricultural Sciences, Shenzhen 518120, China; Shenzhen Branch, Guangdong Laboratory of Lingnan Modern Agriculture, Genome Analysis Laboratory of the Ministry of Agriculture and Rural Affairs, Agricultural Genomics Institute at Shenzhen, Chinese Academy of Agricultural Sciences, Shenzhen 518120, China; Shenzhen Branch, Guangdong Laboratory of Lingnan Modern Agriculture, Genome Analysis Laboratory of the Ministry of Agriculture and Rural Affairs, Agricultural Genomics Institute at Shenzhen, Chinese Academy of Agricultural Sciences, Shenzhen 518120, China; Shenzhen Branch, Guangdong Laboratory of Lingnan Modern Agriculture, Genome Analysis Laboratory of the Ministry of Agriculture and Rural Affairs, Agricultural Genomics Institute at Shenzhen, Chinese Academy of Agricultural Sciences, Shenzhen 518120, China; Shenzhen Branch, Guangdong Laboratory of Lingnan Modern Agriculture, Genome Analysis Laboratory of the Ministry of Agriculture and Rural Affairs, Agricultural Genomics Institute at Shenzhen, Chinese Academy of Agricultural Sciences, Shenzhen 518120, China; Shenzhen Branch, Guangdong Laboratory of Lingnan Modern Agriculture, Genome Analysis Laboratory of the Ministry of Agriculture and Rural Affairs, Agricultural Genomics Institute at Shenzhen, Chinese Academy of Agricultural Sciences, Shenzhen 518120, China; Shenzhen Branch, Guangdong Laboratory of Lingnan Modern Agriculture, Genome Analysis Laboratory of the Ministry of Agriculture and Rural Affairs, Agricultural Genomics Institute at Shenzhen, Chinese Academy of Agricultural Sciences, Shenzhen 518120, China; Shenzhen Branch, Guangdong Laboratory of Lingnan Modern Agriculture, Genome Analysis Laboratory of the Ministry of Agriculture and Rural Affairs, Agricultural Genomics Institute at Shenzhen, Chinese Academy of Agricultural Sciences, Shenzhen 518120, China; Shenzhen Branch, Guangdong Laboratory of Lingnan Modern Agriculture, Genome Analysis Laboratory of the Ministry of Agriculture and Rural Affairs, Agricultural Genomics Institute at Shenzhen, Chinese Academy of Agricultural Sciences, Shenzhen 518120, China; State Key Laboratory of Rice Biology, China National Rice Research Institute, Hangzhou 310006, China; Shenzhen Branch, Guangdong Laboratory of Lingnan Modern Agriculture, Genome Analysis Laboratory of the Ministry of Agriculture and Rural Affairs, Agricultural Genomics Institute at Shenzhen, Chinese Academy of Agricultural Sciences, Shenzhen 518120, China; Shenzhen Branch, Guangdong Laboratory of Lingnan Modern Agriculture, Genome Analysis Laboratory of the Ministry of Agriculture and Rural Affairs, Agricultural Genomics Institute at Shenzhen, Chinese Academy of Agricultural Sciences, Shenzhen 518120, China; Yazhouwan National Laboratory, Sanya 572024, China; Shenzhen Branch, Guangdong Laboratory of Lingnan Modern Agriculture, Genome Analysis Laboratory of the Ministry of Agriculture and Rural Affairs, Agricultural Genomics Institute at Shenzhen, Chinese Academy of Agricultural Sciences, Shenzhen 518120, China; Shenzhen Branch, Guangdong Laboratory of Lingnan Modern Agriculture, Genome Analysis Laboratory of the Ministry of Agriculture and Rural Affairs, Agricultural Genomics Institute at Shenzhen, Chinese Academy of Agricultural Sciences, Shenzhen 518120, China; Shenzhen Branch, Guangdong Laboratory of Lingnan Modern Agriculture, Genome Analysis Laboratory of the Ministry of Agriculture and Rural Affairs, Agricultural Genomics Institute at Shenzhen, Chinese Academy of Agricultural Sciences, Shenzhen 518120, China; Shenzhen Branch, Guangdong Laboratory of Lingnan Modern Agriculture, Genome Analysis Laboratory of the Ministry of Agriculture and Rural Affairs, Agricultural Genomics Institute at Shenzhen, Chinese Academy of Agricultural Sciences, Shenzhen 518120, China; Shenzhen Branch, Guangdong Laboratory of Lingnan Modern Agriculture, Genome Analysis Laboratory of the Ministry of Agriculture and Rural Affairs, Agricultural Genomics Institute at Shenzhen, Chinese Academy of Agricultural Sciences, Shenzhen 518120, China; Shenzhen Branch, Guangdong Laboratory of Lingnan Modern Agriculture, Genome Analysis Laboratory of the Ministry of Agriculture and Rural Affairs, Agricultural Genomics Institute at Shenzhen, Chinese Academy of Agricultural Sciences, Shenzhen 518120, China; Shenzhen Branch, Guangdong Laboratory of Lingnan Modern Agriculture, Genome Analysis Laboratory of the Ministry of Agriculture and Rural Affairs, Agricultural Genomics Institute at Shenzhen, Chinese Academy of Agricultural Sciences, Shenzhen 518120, China; Shenzhen Branch, Guangdong Laboratory of Lingnan Modern Agriculture, Genome Analysis Laboratory of the Ministry of Agriculture and Rural Affairs, Agricultural Genomics Institute at Shenzhen, Chinese Academy of Agricultural Sciences, Shenzhen 518120, China; Shenzhen Branch, Guangdong Laboratory of Lingnan Modern Agriculture, Genome Analysis Laboratory of the Ministry of Agriculture and Rural Affairs, Agricultural Genomics Institute at Shenzhen, Chinese Academy of Agricultural Sciences, Shenzhen 518120, China; National Key Laboratory of Tropical Crop Breeding, Tropical Crops Genetic Resources Institute, Chinese Academy of Tropical Agricultural Sciences, Haikou 571101, China; Shenzhen Branch, Guangdong Laboratory of Lingnan Modern Agriculture, Genome Analysis Laboratory of the Ministry of Agriculture and Rural Affairs, Agricultural Genomics Institute at Shenzhen, Chinese Academy of Agricultural Sciences, Shenzhen 518120, China; Yazhouwan National Laboratory, Sanya 572024, China; State Key Laboratory of Rice Biology, China National Rice Research Institute, Hangzhou 310006, China; Shenzhen Branch, Guangdong Laboratory of Lingnan Modern Agriculture, Genome Analysis Laboratory of the Ministry of Agriculture and Rural Affairs, Agricultural Genomics Institute at Shenzhen, Chinese Academy of Agricultural Sciences, Shenzhen 518120, China; Yazhouwan National Laboratory, Sanya 572024, China

**Keywords:** transposable element, super pan-genome, pan-TE, rice

## Abstract

Transposable elements (TEs) are ubiquitous genomic components and hard to study due to being highly repetitive. Here we assembled 232 chromosome-level genomes based on long-read sequencing data. Coupling the 232 genomes with 15 existing assemblies, we developed a pan-TE map comprising both cultivated and wild Asian rice. We detected 177 084 high-quality TE variations and inferred their derived state using outgroups. We found TEs were one source of phenotypic variation during rice domestication and differentiation. We identified 1246 genes whose expression variation was associated with TEs but not single-nucleotide polymorphisms (SNPs), such as *OsRbohB*, and validated *OsRbohB*’s relative expression activity using a dual-Luciferase (LUC) reporter assays system. Our pan-TE map allowed us to detect multiple novel loci associated with agronomic traits. Collectively, our findings highlight the contributions of TEs to domestication, differentiation and agronomic traits in rice, and there is massive potential for gene cloning and molecular breeding by the high-quality Asian pan-TE map we generated.

## INTRODUCTION

Transposable elements (TEs) are ubiquitous components of genomes and one of the major forces driving genome evolution [1]. As early as 1948, Barbara McClintock observed that transposons were associated with color variegation in maize kernels and leaves [2], revealing the crucial role of TEs in phenotypic variation. TEs also represent a major source of genomic variation. Two classes of TE are roughly distinguished: retroelements and DNA transposons. Retroelements are further classified as long terminal repeats (LTRs, including *Gypsy, Copia* and unknown elements) and non-LTRs (short and long interspersed retrotransposable elements [SINE and LINE, respectively]), and DNA transposons are classified as miniature inverted repeat transposable elements (MITEs, including *Stowaway* and *Tourist*), DTC (*CACTA*), DTA (*hAT*), DTT (*Tc1-Mariner*), DTM (*Mutator*), DTH (*PIF-Harbinger*) and *Helitron* elements [3]. Increasing evidence has supported the hypothesis that TEs are responsible for many well-known human diseases [4] and various plant phenotypes [5–7]. For example, an LTR retrotransposon insertion upstream of an apple MYB transcription factor, *MdMYB1*, a core transcriptional activator of anthocyanin biosynthesis, is associated with the red-skinned phenotype of apples [8]. An insertion of a *Gret1* LTR retrotransposon in the Chardonnay grape variety, which is not present in the Cabernet variety, generated a loss-of-function allele of the *Vvmby1A* gene, leading to a loss of color in the fruit [9]. A TE inserted in a regulatory region of the maize domestication gene (*tb1*) acts as an enhancer of gene expression and partially explains the increased apical dominance in maize compared to its progenitor, teosinte [10].

Recently reported crop TE variation data sets mainly consist of TE variations obtained from Illumina short-read sequencing [5,7,11], such as one constructed using short-read sequences from 524 *Brassica rapa* accessions [5] and one constructed using short-read sequences from 602 tomato accessions [7]. However, identifying highly repetitive sequences such as TEs using short-read sequencing data is difficult and predictions have low accuracy, especially for some TE families that have a small length and a very high copy number, such as the SINE, LINE and MITE families. Identification of TE variations using high-quality genome sequences based on long-read sequencing data is the most accurate method to identify TE variations. At present, a population-scale TE variation data set based on de novo long-read assemblies has been published only in *Drosophila nasuta* [12].

Rice is a major food crop for half of the global population [13,14], and improving rice productivity is essential for meeting the growing demands of the ever-increasing world population [15,16]. Asian cultivated rice (*Oryza sativa, Os*) was domesticated from Asian wild rice (*Oryza rufipogon, Or*) and has two main types: Geng (*Oryza sativa japonica, Osj*) and Xian (*Oryza sativa indica, Osi*) [17]. TEs are known to contribute to phenotypic variation in rice populations. For instance, a *Helitron* in the *MYB61* [18] promoter was reported to affect rice nitrogen utilization; an LTR insertion in the *GY3* promoter suppresses its expression and results in a higher content of active cytokinins in young panicles [19]; and the *PIF-Harbinger* transposon-derived gene *PANDA* epigenetically coordinates panicle number and grain size in rice [20]. Identification of TEs across rice accessions would facilitate efforts to determine the contribution of TEs to expression and phenotypic variation. Three studies of population-scale TE variations based on Illumina short-reads [11,21,22] have been published in rice. However, these studies mainly focused on *Os* accessions, with *Or* and domestication-associated TE variations remaining underexplored. In addition, although an increasing number of rice studies based on high-quality assemblies have been published, population-level, high-quality studies of TE variations have not been reported. Moreover, because of the absence of rice outgroup accessions in previous studies, it is hard to distinguish the ancestral and derived state of TE variations. Thus, building a pan-TE resource using high-quality chromosome-level assemblies and inferring whether TE variations are ancestral or derived based on outgroups are desirable, and such information will help us to know the true extent of TE variation in plants and their biological impacts on rice environmental adaptation and domestication.

Here, we de novo assembled chromosome-level genomes for 232 diverse rice accessions by integrating Oxford Nanopore Technology (ONT) long-read data and Illumina short-read data from public databases. Combining these assemblies with 15 existing rice assemblies, we constructed a pan-TE map of 247 Asian accessions (218 *Os* and 29 *Or* accessions) and inferred whether TE variations were ancestral or derived using three non-Asian accessions as outgroups (*Oryza glaberrima, Oryza barthii* and *Oryza glumaepatula*). We analyzed TE variation characteristics and the role of TE mobilization in rice domestication diversification. Moreover, we analyzed the contribution of TE variation to gene expression variation and identified 1246 genes whose expression levels were significantly associated with TE variation but not single-nucleotide polymorphisms (SNPs). Importantly, our pan-TE map enabled genome-wide association study (GWAS)-based identification of loci associated with phenotypes (i.e. seed setting rate under cold stress), which were undetectable when using only SNPs. Taken together, the pan-TE map is a valuable resource for understanding the genetic basis of environmental adaptation, domestication and agronomic traits in rice.

## RESULTS

### De novo assembly and pan-TE map construction for Asian rice accessions

**Figure 1.**
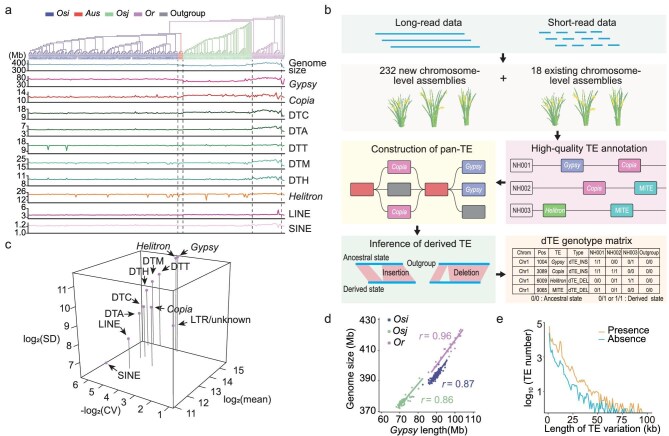
Construction of a pan-TE map and evaluation of TE variations in Asian accessions using chromosome-level genomes. (a) Landscape of genome size and TE content across different subpopulations. Phylogeny of 250 accessions were based on whole-genome SNPs (top); accessions in different subpopulations are indicated by different colors. *Osi, Aus, Osj, Or* and outgroup respectively refer to *O. sativa indica, O. sativa aus, O. sativa japonica, O. rufipogon*, and three non-Asian accessions (one *O. glaberrima*, one *O. barthii* and one *O. glumaepatula*). Length of genome and TE content (Mb) in each genome are indicated (bottom). The length of *Gypsy, Copia*, DTC, DTA, DTT, DTM, DTH, *Helitron*, LINE and SINE elements in each genome are indicated. (b) Overview of the pipeline for pan-TE map construction. Firstly, 232 high-quality chromosomal-level assemblies were de novo assembled by integrating public long-read and short-read data. After combining the de novo assemblies with 18 existing assemblies, the TE sequences were annotated and a TE library was generated. To construct a pan-TE map, the TE variations were identified by combing the results of genome alignment, the TE library and long-read data. Subsequently three non-Asian accessions were used as outgroups (henceforth ‘outgroup’) to infer whether a given TE variation was in derived state or ancestral state in each accession. A TE variation that has both derived state and ancestral state in Asian rice accessions was defined as derived TE variation (henceforth ‘dTE’). Ancestral state indicates that the genotype of the locus in a given accession (0/0) is the same as that of the outgroup (0/0); derived state indicates that the genotype of the locus in a given accession (1/1 or 0/1) is different from that of the outgroup (0/0), including homozygous (1/1) and heterozygous (0/1) genotype. Finally, a dTE genotype data set in matrix format is generated for use in downstream analysis, including domestication, gene expression and GWAS. (c) The copy number variation for different TE families in 250 natural accessions. The x axis represents the copy number variation for each TE family across accessions, evaluated as coefficient of variation (CV); the y axis represents the average number of TEs in each family; the z axis represents the differences in total TE number for each family among accessions in total TE number, evaluated as standard deviation (SD). (d) Pearson correlation coefficients for comparisons between total length of *Gypsy* elements and genome size across different subpopulations. Colored dots and lines indicate data from each subpopulation. (e) Length distributions of TE variations in the non-redundant TE data set for Asian accessions.

We collected the relevant long-read and short-read data for 250 rice accessions, chromosomal-level assemblies for 18 accessions, and leaf transcriptome data for 202 *Os* accessions, which were selected based on the geographical distribution and genetic diversity of rice from public databases (Table S1a, see ‘Materials and data collection’). The 250 accessions included 247 Asian rice accessions and 3 non-Asian rice accessions (1 *O. glaberrima*, 1 *O. barthii* and 1 *O. glumaepatula*) as outgroups (henceforth termed ‘outgroups’) (Table S1a). A phylogenetic tree constructed using whole-genome SNP data for the 250 accessions clearly divided these materials into four main subpopulations, *Osi, Aus, Osj* and *Or* (Fig. 1a). Since data for only five *Aus* accessions were collected, which is not enough for population analysis, they were not included in subsequent subpopulation analysis. Finally, 146 *Osi*, 61 *Osj* and 27 *Or* were retained to represent the *Osi, Osj* and *Or* subpopulations for population comparison analysis. The results of admixture and principal component analysis (PCA) were highly consistent with those of the phylogenetic tree (Fig. S1a–c).

We found that a genome has an average of 28 895 intact TEs (Fig. S1d), and the number of intact TEs of chromosomal-level genomes is more than that of contig-level genomes (Fig. S1d), thus, obtaining high-quality chromosomal-level genomes was critical for constructing a pan-TE map. To this end, the non-chromosome-level assemblies of 232 accessions were de novo assembled with ONT long reads using NextDenovo (version 2.4) [23] (Fig. 1b), and the assemblies were corrected with both long reads and Illumina short reads using Nextpolish (version 1.4.0) [24]. The 232 new assemblies had an average length of 390.7 ± 11.3 Mb and average contig N50 of 23.7 ± 6.0 Mb (Table S1b). An average of 99.7% ± 0.2% of contigs were anchored to the chromosomes (Table S1b). We next evaluated the quality of the new assemblies and found that: (i) an average of 98.5% ±  0.5% of Illumina short reads could be mapped to their corresponding assembly, which is similar to the mapping rate (98.0%) obtained for Nipponbare (NIP) [15,25] (Table 1b and Fig. S1e); (ii) the assembly quality was comparable to that of the NIP genome [15,25], with 97.9% ±  0.2% of a Benchmarking Universal Single-Copy Orthologs (BUSCO) [26] 1440 reference gene set captured in each new assembly (Table S1b and Fig. S1e); and (iii) the average accuracy at the single-base level was high (99.9996% ± 0.0004%) for the new assemblies (Table S1b). In addition, the high-throughput chromosome conformation capture (Hi-C) data from four assemblies (Fig. S1f) indicated the high continuity and completeness of the new assemblies. These results together suggested that the quality of all 232 new assemblies was comparable to, or better than, the levels achieved for the NIP reference genome [25], highlighting the high-quality of these new assemblies.

Next, DNA repeat sequences were annotated in the 250 high-quality genomes consisting of the 232 new assemblies and 18 existing chromosome-level assemblies downloaded from previous reports [27–31] using Extensive de-novo TE Annotator (EDTA) [32]. An average of 199.2 Mb of TEs per assembly was annotated, accounting for an average of 51.0% of the total assembly length (Table S1c); overall, these values are consistent with a previous report [15]. The LTR assembly index (LAI) [33] was used to evaluate the integrity of the TE sequences in these new assemblies, and the average LAI per assembly was 21.3 ±  3.9 (Table S1b and Fig. S1g), which reached the ‘gold standard’ level (LAI > 20). Furthermore, comparison of the NH231 (9311) assembly sequence with bacterial artificial chromosome (BAC) clones [34] sequenced using Sanger technology showed that both sequences were of similar quality in the TE regions (Table S1d and Fig. S1h–i).

We found that the accessions were highly diverse in terms of TE numbers. The coefficient of variation and standard deviation for the numbers of TEs per family across all 250 accessions were 0.01–0.58 and 70–3257, respectively (Fig. 1c; Table S1c). The variation in the total number of TEs across these 250 accessions allowed us to study the impacts of TEs on rice evolution. For example, in our previous study we found that the observed variation in genome size can be primarily explained by the number of LTRs [11]. Using our present TE data sets for *Osi, Osj* and *Or* accessions, we further found that genome size variation in Asian rice was mainly due to the variation in the number of *Gypsy* elements (Fig. 1d; Fig. S1j).

To survey the landscape of TE variation in Asian rice, we compared 250 chromosomal-level assemblies against the Nipponbare assembly using minimap2 (version 2.24-r1122) [35]. Presence/absence variants (PAVs, >50 bp) were identified using custom perl scripts. When >90% of the sequences of a PAV are TE sequences, we consider the PAV as a TE variation (or TE-derived PAV) (Fig. S1k) [36]. A pan-TE map was developed eventually (total 177 084 non-redundant TE variations, Fig. 1b). To quantitatively estimate the accuracy of our pan-TE map, we manually examined 200 randomly selected TE variations by visualizing the corresponding long-read mapping using the Integrative Genomics Viewer Browser. Using this method, we estimated the accuracy of our pan-TE map to be 97.5% (Table S1e). We compared the TE variations identified in *Os* accessions with those previously detected using short-read sequences from the 3K-RG data set [11], and found that the average length of TE variation we identified in the 218 *Os* accessions (3813 bp) was larger than that of the 3K-RG data set (346 bp) [11,21]. Notably, almost half the TE variations were relatively long (the lengths of 47.3% TE variations were longer than 1 kb) (Fig. 1e), and 14.0% of TE variations contained at least two TE sequences. Together, these results suggested that using high-quality assemblies improved the identification of TE variations.

### Inference and characterization of TE variations representing a derived state in Asian rice

**Figure 2.**
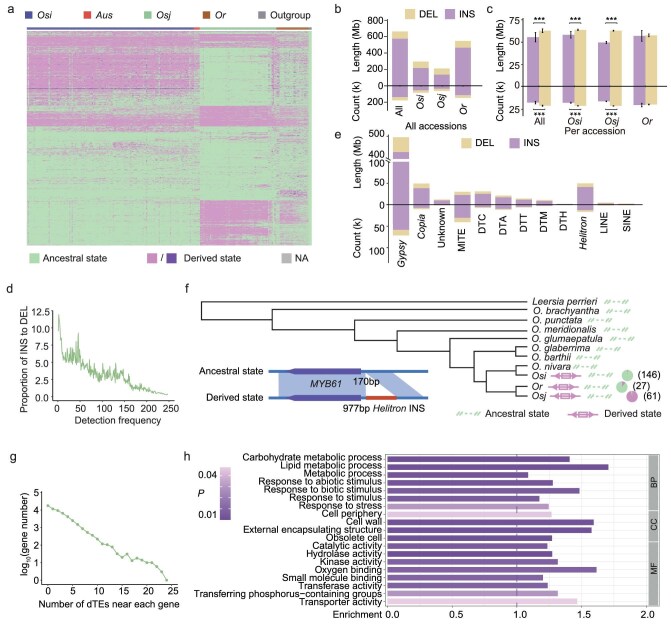
Inference and characterization of TE variations representing a derived state across Asian accessions. (a) The landscape of dTEs across the Asian accessions. Three non-Asian accessions were used as outgroups (henceforth termed ‘outgroup’) to infer whether a given TE variation was in derived state or ancestral state in each accession. The TE variations, which have both derived state and ancestral state in Asian rice accessions, were defined as derived TE variations (henceforth ‘dTEs’). Ancestral state indicates that the genotype of a TE variation in an accession is consistent with that of the outgroup; derived state indicates that the genotype of a TE variation, homozygous (left) or heterozygous (right), in an accession is different from that of the outgroup. NA indicate that the genotype is uncertain. *Osi, Aus, Osj, Or* and outgroup respectively refer to *O. sativa indica, O. sativa aus, O. sativa japonica, O. rufipogon* and three non-Asian accessions (*O. glaberrima, O. barthii* and *O. glumaepatula*). (b) Total length and number of non-redundant dTEs and sequences detected across different subpopulations. DEL and INS refer to deletion and insertion events identified by comparing the Asian accessions to the outgroups, respectively. ‘All’ refers to all the Asian rice accessions in the present study. (c) Average length and number of dTEs for each accession across different subpopulations. ***P < 0.001, significance was determined using the Student's *t*-test. (d) The count ratio of INS events to DEL events for different frequencies of dTEs in Asian rice accessions. (e) Total length and number of dTEs for TE families in Asian rice accessions. (f) A *Helitron* in the *MYB61* promoter region was found in all *Osj* accessions in a previous study, but was undetectable in *Or* and *Osi* accessions and the rice outgroup genomes CC and EE. However, the *Helitron* inserted in some *Or* and *Osi* accessions in our high-quality Asian pan-TE map. (g) Distribution of the number of dTEs near each gene (within ±2 kb of a gene body). (h) Gene ontology (GO) analyses of the genes containing several dTEs (‘the genes’ were ranked by the number of dTEs overlapping with their genic region, and the top 5% of ranked genes were used in the GO analyses). BP, CC and MF respectively refer to biological process, cellular component and molecular function.

In previous studies [11,22] of population-scale TE variations, there were no rice outgroup accessions, making it hard to distinguish whether a given accession was with the ancestral state or the derived state in a given TE variation. In addition, without outgroups, it was not possible to determine whether each TE variation was the result of a TE insertion (INS) or deletion (DEL) event. Since all three outgroups, including one *O. glaberrima* [15], one *O. barthii* [15] and one *O. glumaepatula* (https://www.ncbi.nlm.nih.gov/nuccore/CM002522.2), are ancestral relatives to Asian rice, and there has been no introgression between them [37,38], we were able to use them as outgroups to represent the ancestral states of TE variations, and to infer the state of each TE variation in each accession. TE variations that have both derived state and ancestral state in Asian rice accessions were defined as derived TE variations (henceforth, dTEs). Derived state indicates that the genotype of the TE variation in a given accession is different from outgroups, while ancestral state indicates that the genotype of the TE variation in a given accession is the same as outgroups (Figs 1b and 2a). We found 169 798 non-redundant dTEs (95.8% of non-redundant TE variations) in the Asian accessions with a total length of 647.9 Mb (Fig. 2b), which was ∼3.5-fold higher than the total length of TE sequences in NIP (185.4 Mb). Most of the non-redundant dTE sequences were contributed by *Or* (Fig. 2b), possibly because of the reference bias using Nipponbare as the reference and higher genetic diversity of *Or* compared with *Osi* and *Osj*. An obviously higher number of dTE INS events compared with DEL events was observed in the Asian rice population (Fig. 2b). However, except for *Or* accessions, the number/length of dTE DELs in each accession was significantly higher than that of dTE INSs in each accession (Fig. 2c, *P* < 0.001). We speculate that this might be due to the unbalanced frequency between dTE INS and DEL events in Asian rice populations. 99 840 (72.9%) dTE INSs and 10 258 (31.3%) dTE DELs were present in one or only a few accessions (present frequency < 0.05) (Fig. S2a). When comparing the ratio between INSs and DELs over present frequency of dTE variation, we found a decreased curve (Fig. 2d; Fig. S2a–b), which suggests that the clearing speed to dTE INS and DEL might be different in rice evolution, with a faster clearing speed to dTE INS.

By further analyzing the dTE numbers and lengths per TE family, we found that the majority of dTEs were *Gypsy, Helitron* and *Copia* elements (Fig. 2e), which was consistent with the high proportion of these elements in each genome (Fig. 1a). The inference of dTEs can help reveal the direction and biological significance of natural and/or artificial selection acting on TEs. For example, a 977 bp dTE (*Helitron*) INS near the promoter of a transcription factor gene, *MYB61*, was reported to regulate nitrogen utilization and biomass production in rice, and the *Helitron* INS was found in *Osj* accessions, but not *Or* or *Osi* accessions or the genomes of the CC and EE outgroups in rice in a previous study [38]. However, with our high-quality Asian pan-TE map, we found the *Helitron* INS in some of the *Or* and *Osi* accessions (Fig. 2f), which indicated that the *Helitron* INS occurred before rice domestication. In addition, we found that the *Helitron* sequence varied among Asian accessions (Fig. S2c). Notably, some *Osj* accessions had two copies of this *Helitron* INS, and the two copies were close together (∼145 kb). Because the dTE contains a structurally intact *Helitron* sequence, we estimated the *Helitron* insertion time for each copy in accessions with this dTE and found that these *Helitron* sequences were relatively young overall (Table S1g).

In addition, we calculated the number of dTEs near each gene (within ± 2 kb of a gene body) in our Asian pan-TE map and found that 31.8% of genes were conserved across all Asian accessions (no nearby dTEs were found), while almost half the genes (47.4%) contained two or more dTEs (Fig. 2g). Gene ontology (GO) analyses showed that genes containing no dTEs were significantly enriched in pathways related to basic metabolism and biosynthesis of metabolites (*P <* 0.05), including cell death, photosynthesis, gene expression, amide biosynthetic process and metabolic process (Fig. S2d and Table S1h). In contrast, genes containing several dTEs (i.e. the number of nearby dTEs ranked in the top 5% of all genes in NIP) were significantly enriched for abiotic and biotic response genes (*P* < 0.05), such as resistance genes (*Xa1* and *Pita*) and heat shock protein genes (*OsSHSP2* and *OsHSP1*; Fig. 2h; Table S1h), indicating that TE variations may play important roles in environmental adaptation in nature. This speculation is also supported by a significant negative correlation between latitude and the number of dTEs in each Asian rice accession (Fig. S2e–f).

### Contribution of TE variations to rice domestication and differentiation

**Figure 3.**
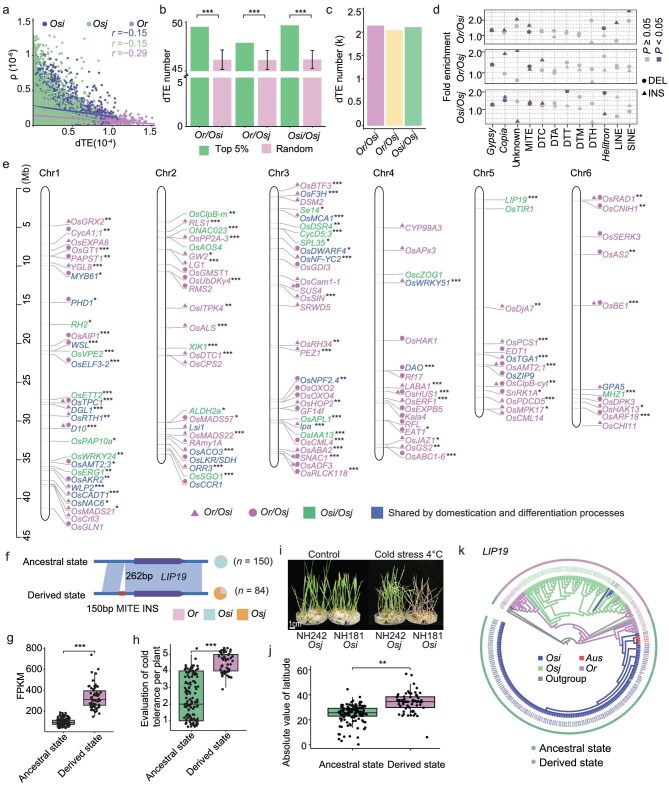
Contribution of dTEs to rice domestication and differentiation. (a) Pearson correlation coefficient between dTE average pairwise diversity (π) and recombination rate (ρ) across different subpopulations. (b) Comparisons of the average numbers of dTEs in the selective windows (i.e. ranked in top 5% of 100 kb *F*_ST_ windows for SNPs) and in windows randomly selected from each permutation for 500 independent permutations between subpopulations. (c) Number of dTEs showing signatures of selection (henceforth ‘selected dTEs’) between *Or* and *Osi, Or* and *Osj*, and *Osi* and *Osj. F*_ST_ outliers based on TE variations were indicated as loci showing signatures of selection. (d) Fold enrichment of selected dTEs in each TE family by the Fisher's exact test. Unknown refers to LTR/unknown family. (e) Positions of functional genes harboring selected dTEs in their genic regions on chromosomes 1–6. For chromosomes 7–12 see Fig. S3g. Genic regions included the gene body, regions of 2 kb upstream (henceforth ‘promoter’) and 2 kb downstream (henceforth ‘downstream’) of the gene body. Functional genes harboring selected dTEs in rice domestication and differentiation processes are displayed by different colored fonts. Differences in the gene expression between subpopulations were tested using the Student's *t*-test. ***P < 0.001, **P < 0.01 and * *P* < 0.05. (f) A 150 bp dTE (*Tourist* MITE) insertion occurred at 262 bp upstream of *LIP19* in *Osj* accessions. (g–h) Differences in the expression level of *LIP19* (g) and cold tolerance (h) between *Osi* accessions with the ancestral state (*n* = 131 and *n* = 122, respectively) and *Osj* accessions with the derived state (*n* = 58 and *n* = 54, respectively) of the 150 bp dTE INS. Significance was tested by the Student's *t*-test, ***P < 0.001. (i) Phenotypes of NH242 (an *Osj* accession) and NH181 (an *Osi* accession) under control (left) and cold stress treatments (right). For the cold stress treatment, 10-day-old seedlings were transferred to 4°C for 72 h and recovered for 4 days in a greenhouse at 30°C. (j) Differences in the absolute value of latitude of the accessions with this dTE INS (derived state) and those without this dTE INS (ancestral state) near *LIP19.* Significance was tested by the Wilcoxon-Mann-Whitney test, **P < 0.01. (k) Phylogenetic tree based on the sequence alignments of the promoter and gene body regions of *LIP19* across all accessions in the present study. Accessions in different subpopulations are indicated by different colors.

TEs can accumulate in regions of low recombination due to the action of Hill-Robertson interference [39], as we found a significant negative correlation between dTE diversity and the recombination rate of genomes in rice (Fig. 3a; Fig. S3a). However, we found a significantly positive correlation between *Gypsy* diversity and the recombination rate of genomes in *Osi* accessions (Fig. S3b), and the differences in the correlation directionality across TE types and subpopulations have been reported in other plants [9,39], indicating that the INSs and DELs of TE variations are a potentially rich source of variation in plant adaptive evolution. Numerous plant domestication and agronomic traits have been associated with particular TE variations, thus we speculated that dTEs may play an important role in evolutionary processes, such as domestication and differentiation in rice. To explore the impacts of TE variations on the domestication and differentiation of Asian rice, we investigated TE variations in 247 diverse Asian rice accessions. A phylogenetic tree constructed using our dTE data set for the Asian rice accessions clearly divided the accessions into the groups of *Osi, Osj, Aus* and *Or* (Fig. S3c), consistent with the SNP-based rice phylogeny (Fig. 1a). Meanwhile, the results of admixture (Fig. S3d) and PCA (Fig. S3e–f) using the dTE data set also revealed that TE variations were closely associated with Asian rice domestication and differentiation.

Next, we calculated the average number of dTEs in the selective windows (i.e. ranked in the top 5% of 100 kb *F*_ST_ windows for SNPs) between subpopulations. dTEs were significantly enriched in selective windows than expected at random, in comparisons between *Or* and *Osi, Or* and *Osj*, and *Osi* and *Osj*, even under the conditions in which we controlled the sample size of each subpopulation to the same level (Fig. 3b, *P* < 2.2e-16; Fig. S3g), indicating that dTEs probably participated in rice domestication (from *Or* to *Osi* and *Or* to *Osj*) and differentiation (between *Osi* and *Osj*). Thus, to determine whether the observed differential distribution of dTE genotypes among subpopulations was due to selection, we estimated the divergence of each dTE across the genome using *F*_ST_ analysis, and 2141 and 2043 of the dTEs showing signatures of selection (henceforth ‘selected dTEs’; i.e. locus showing signatures of selection, i.e. *F*_ST_ outlier between *Or* and *Osi* [outlier = 0.85], *Or* and *Osj* [outlier = 0.84]) were detected in the *Or*-*Osi* and *Or*-*Osj* comparisons, respectively (Fig. 3c). In total, 3935 selected dTEs possibly involved in the domestication processes were ultimately identified (Fig. 3c). Using the same *F*_ST_ analysis approach, we also identified 2108 selected dTEs possibly involved in differentiation between *Osi* and *Osj* (outlier *F*_ST_ value = 0.94) (Fig. 3c). Almost all LTR types and two DNA types, MITE and *Helitron*, were significantly enriched in rice domestication and differentiation processes (*P* < 0.05, Fig. 3d), while SINE and LINE were significantly enriched in the domestication processes from *Or* to *Osi* (*P* < 0.05, Fig. 3d).

We analyzed the genes overlapping with selected dTEs and identified 3860 potential candidate genes (henceforth ‘dTE-genes’), including 2992 dTE-genes with evidence of selection between *Or* and *Os* (1634 dTE-genes between *Or* and *Osi*, and 1580 dTE-genes between *Or* and *Osj*) and 1750 dTE-genes with evidence of selection between *Osi* and *Osj*. Of these, we identified the selection and expression differences for 169 genes with known functions (Fig. 3e; Fig. S3h). We found selected dTEs near previously identified putative domestication genes, such as *An-2* [40]. In addition, we found a selected dTE (345 bp) INS in the intron of a known gene, *OsAKR2*, between *Or* and *Osj*, which was located in a region in strong linkage disequilibrium (LD) with the known domestication gene *qSh1* (Fig. S3i). We can infer that this dTE INS may be also strongly selected in the domestication process due to strong linkage disequilibrium with the causal SNP variant in *qSh1.* We also found a 112 bp selected dTE (LINE) DEL in the 3′UTR of the drought tolerant gene *OsCML4* in upland *Osj* accessions, and the expression level of *OsCML4* in the upland *Osj* accessions with the dTE DEL was significantly higher than that of the *Or* accessions without this dTE DEL (Fig. S3j). Increased *OsCML4* expression likely causes the upland *Osj* accessions to have stronger drought resistance than the other *Os* accessions.

We found two dTE-genes that might be involved in environmental adaptation, promoting the formation of diverse crops during the differentiation of *Osi* and *Osj*. For example, a selected dTE occurred between *Osi* and *Osj* subpopulations (*F*_ST_ = 0.94, rank: 4.42%). This 157 bp sequence with a *Helitron* was located downstream of a known heat stress gene, *OsClpB-m* [41]. It was found in the outgroups (ancestral state), some of the *Or* accessions (18 out of 27; the genotype of one accession was uncertain), and most of the *Osi* accessions (120 out of 146; the genotypes of 21 accessions were uncertain), but absent in most of the *Osj* accessions (derived state; 54 out of 61; the genotypes of the remaining 7 accessions were uncertain) (Fig. S3k). The *Osj* accessions with the dTE DEL event had significantly lower expression levels of *OsClpB-m* [41] (Fig. S3k, *P* < 0.01) and heat tolerance (Fig. S3k, *P* < 0.001) than the O*si* accessions without the dTE DEL event.

Another dTE-gene example was the rice cold-induced *LIP19* [42] gene, which encodes a 148-amino-acid basic region/leucine zipper protein and is highly expressed in the mesophyll cells surrounding vascular bundles of rice plants. We detected a 150 bp selected dTE (*Tourist* MITE, *F*_ST_ = 0.95, rank: 3.71%) inserted in the promoter of *LIP19* (Fig. 3f). The 150 bp region was present in almost all *Osj* accessions (60 out of 61) but few *Osi* accessions (4 out of 146, Fig. 3f). The expression level of *LIP19* in the *Osj* accessions with the dTE INS was shown to be significantly higher than those *Osi* accessions without the dTE INS (Fig. 3g, *P* < 0.001). It is therefore possible that this dTE INS has contributed to cold tolerance in the *Osj* accessions rather than those *Osi* accessions without the dTE INS (Fig. 3h–i, *P* < 0.001). *Osj* accessions are distributed in the regions with a higher latitude and colder temperature, and the dTE state (including derived state and ancestral state) of the temperature adaptation genes also distributed according to the latitude (Fig. 3j; Fig. S3l), which may be affected by climate adaptability selection due to temperature. The phylogenetic tree of the dTE and *LIP19* sequences from the 247 Asian rice accessions and three outgroups showed that the outgroups were without the 150 bp dTE region. The *Osj* accessions and *Osi* accessions were grouped with different *Or* accessions, which was consistent with the distribution of the dTE INS event. The dTE in the *Osj* subpopulation was derived from *Or* accessions (Fig. 3k). The fact that the distribution of this dTE INS event was nearly fixed in the *Osj* subpopulation (60 out of 61) and nearly totally absent in the *Osi* subpopulation (4 out of 146), demonstrated that the dTE was selected during the domestication of *Osj* and *Osi*, potentially because of its effect on cold response through the modulating expression of the *LIP19* gene. These results suggested that dTEs probably contribute to climate adaptability by altering gene expression in rice.

### TE variations associated with gene expression

**Figure 4.**
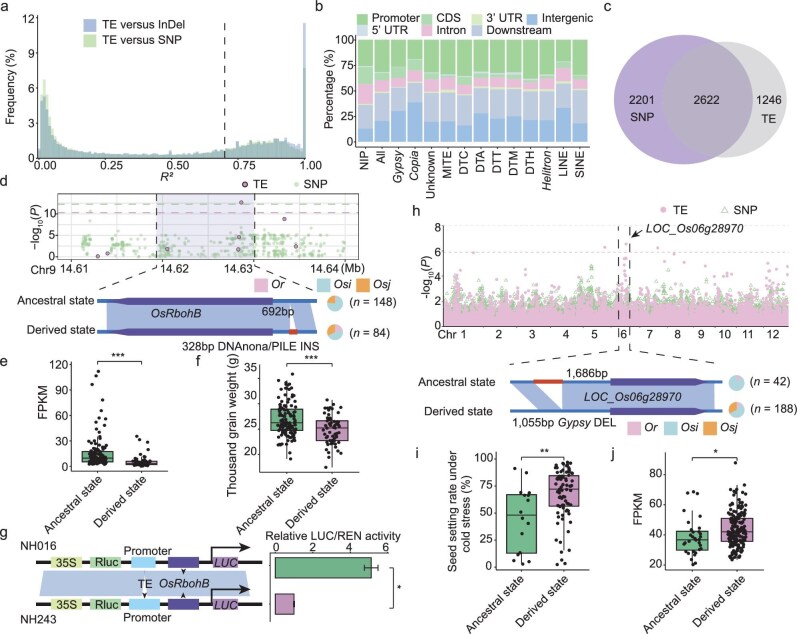
TE variations associated with gene expression and agricultural traits. (a) The distribution of linkage disequilibrium values (LD, *R²*) between dTEs and SNPs/InDels within 50 kb of the dTEs. For each dTE, the maximum *R²* with adjacent SNPs/InDels (within 50 kb on either side) was recorded. The dashed line indicates *R²* = 0.70. (b) Percentage of dTEs overlapping with genic regions. Genic regions include gene body (CDS, 3’UTR, 5’UTR and intron), and the regions within 2 kb upstream (henceforth ‘promoter’) and 2 kb downstream (henceforth ‘downstream’) of the gene body. Unknown refers to LTR/unknown family. ‘All’ refers to the percentage of dTEs in the genic regions of all dTE loci identified in the present study, and ‘NIP’ refers to the percentage of TEs overlapping with genic regions in the Nipponbare genome. (c) Number of eGenes associated with dTE and SNP variants. eGenes are genes whose expression is significantly associated with dTE and SNP variants. (d) Manhattan plot of *OsRbohB* expression level and the dTE variants and SNPs (top). The leading dTE (328 bp) insertion (INS) associated with *OsRbohB* expression level is indicated (bottom). (e–f) Differences in the expression level of *OsRbohB* (e) and the thousand grain weight (f) between accessions with the ancestral state of the dTE INS event (*n* = 125 and *n* = 121, respectively) and those with the derived state (both *n* = 61). Significance was tested by the Student's *t*-test, ***P < 0.001. (g) Transcriptional activation assays by contransfecting rice protoplasts. Error bars represent the mean ± SD of three biological replicates. (h) GWAS for seed setting rate under cold stress using the TE and SNP data sets for the *Os* accessions. A locus on chromosome 6, which was identified by TEs but not by SNPs, was significantly associated with seed setting rate under cold stress. The triangles represent SNPs and dots represent dTEs. The most strongly associated dTE was a 1.0 kb dTE (*Gypsy*) deletion (DEL) event in the promoter of *LOC_Os06g28970*. (i and j) Comparison of seed setting rate under cold stress (i) and the expression level of *LOC_Os06g28970* (j) between accessions with (i.e. derived state, *n* = 16 and *n* = 32, respectively) and without (i.e. ancestral state, *n* = 83 and *n* = 169, respectively) the dTE DEL event. Significance was tested by the Student's *t*-test, **P < 0.01, *P < 0.05.

In many organisms, including plants and animals, TEs have been shown to be associated with differences in expression level [43]. To assess more systematically whether TEs are a potentially important source of gene expression variation [5,12,22], we first measured the LD of dTE with nearby SNPs and insertion-deletion polymorphisms (InDels). We observed that a large fraction of dTEs are in strong LD (*R*^2^ > 0.7) with adjacent (50 kb on either side) SNPs and InDels (43.4% and 47.6%, respectively, Fig. 4a), and a small proportion (7.5% and 11.3%, respectively) are in complete LD (*R*^2 ^ = 1). However, nearly one-fourth of dTEs are in weak LD (25.2% and 30.3%, respectively, *R*^2  ^< 0.1), indicating that TE variations are not perfectly tagged by SNPs and InDel markers. We next calculated the number of dTEs in terms of relative position to genes and found that 79.5% of dTEs overlap with genic regions (within ± 2 kb of a gene body) (Fig. 4b). Compared with the distribution of TEs in the NIP genome [25], the dTEs from the Asian pan-TE map were significantly enriched in transcriptional regulatory regions (*P* < 0.05) and depleted in coding sequences (CDSs, *P* < 0.001) and intronic regions (*P* < 0.001, Fig. 4b). All TE families showed a pattern similar to that of the whole dTE data set (Fig. 4b), indicating that TEs may play a role in regulating gene expression.

To estimate the relative contribution of TE variations and SNPs to gene expression changes, we associated dTEs and SNPs with gene expression levels among *Os* accessions, respectively, and identified expression quantitative trait loci (eQTLs). Using leaf transcriptome data from our previous study [15], we identified 3868 genes whose expression was significantly associated with dTE (referred to as eGenes, Fig. 4c). Of these eGenes, 2622 were also identified in the SNP-eQTL analysis. For example, a dTE (DTM) INS present in the intron of a shared gene *OsGluRS* [44] was found in 60 out of 61 *Osj* accessions, while the leading SNP variant with *OsGluRS* [44] gene expression variations was ∼4.8 kb away from the gene (Fig. S4a), indicating that the dTE is potentially a crucial variation altering *OsGluRS* [44] expression. Knockdown lines of *OsGluRS* [44] in *Osj* accessions resulted in an abnormal phenotype characterized by yellow-green leaves when plants were grown at 26°C or higher (non-permissive temperature) [44]. We also found similar results at the population level; accessions with the dTE INS (the derived state) showed significantly lower expression and lower heat resistance than those without the dTE INS (the ancestral state, Fig. S4a). Another example is a known positive regulator gene of bacterial blight resistance, *OsRP1L1* [45]. A 1.0 kb dTE (*Helitron*) INS in the gene promoter was identified in most *Osj* accessions (57 out of 61) and a few *Osi* accessions (13 out of 146) (Fig. S4b). The expression of *OsRP1L1* was previously shown to ensure appropriate bacterial blight resistance in rice [45]. We found that the accessions with the dTE INS had significantly higher expression levels and were possibly associated with lower susceptibility to bacterial blight than the accessions without the dTE INS (Fig. S4b), indicating that this dTE may play a role in increasing bacterial blight resistance. For the 2622 shared eGenes between TE-eQTL and SNP-eQTL analysis, we compared the expression variance explained by the most significantly associated dTEs and SNPs. For ∼48.9% of these genes, the leading associated dTEs could explain more expression variance than the leading associated SNPs.

Importantly, the expression levels of 1246 eGenes were only associated with dTEs and not associated with SNP data (Fig. 4c), demonstrating the importance of considering dTEs in gene expression. For example, the expression of a rice NADPH oxidase gene, *OsRbohB* [46], was significantly associated with a 328 bp dTE (DNA TE PILE) INS event in its promoter region (Fig. 4d). We did not find any significant association of *OsRbohB* expression with SNPs. The dTE INS was associated with decreased expression of *OsRbohB* in the accessions with the dTE INS (Fig. 4e), and the accessions with the dTE INS had reduced thousand grain weight compared with those without the dTE INS (Fig. 4f) in both the *Osi* and *Osj* accessions (Fig. S4c). It seems plausible that this dTE INS may contribute to thousand grain weight, consistent with the phenotype of the *OsRbohB* mutant showing lower thousand grain weight in a previous study [46]. Compared to the accessions (such as NH016) without the dTE INS, the accessions (such as NH243) with the dTE INS in a *OsRbohB* promoter fragment significantly reduced its relative expression activity (Fig. 4g). These results validated the incomplete LD between dTEs and small variants (SNPs and InDels) and highlighted the potential of dTEs to explain additional variation in gene expression and agronomic traits.

### TE variations associated with agronomic traits

To demonstrate how the pan-TE map can be used to facilitate the identification of agronomic traits associated with TE variations, we performed a GWAS of agronomic traits in *Os* accessions. A *GW5* peak was previously detected in a GWAS on grain width using SNPs or TE variations genotyped using short-read data [47]. We also detected the same signal for grain width. In addition, we identified a new locus on chromosome 7 harboring a 131 bp dTE (*Stowaway* MITE) INS that was significantly associated with grain width (Fig. S5a). The locus was not identified using SNPs or the previous TE variations based on short-reads data. Rice accessions with the dTE INS (derived state) were shown to have significantly lower grain width than those without the dTE INS (ancestral state) (*P* < 0.001, Fig. S5b). Within a 5 kb region of the TE peak, there were two candidate genes. One of them was *LOC_Os07g41000* [48], a gene previously reported to regulate grain width (Fig. S5b–c), indicating that the dTE might control grain width by influencing *LOC_Os07g41000* [48].

Using phenotypes related to heat tolerance to conduct TE-GWAS, we identified two dTE peak loci strongly associated with heat stress in *Osi* accessions; these loci were not identified using the SNP data set of *Osi* accessions (Fig. S5d–g). From the candidate genes near each dTE peak (5 kb on either side), we found a 263 bp dTE (*Gypsy*) DEL, which was associated with the level of heat resistance, and was located near an OsFBX121 F-box domain gene, *LOC_Os04g11890* (Fig. S5d–e, *P* < 0.001). F-box proteins play important roles in responses to environmental stimuli [49], indicating that the dTE might affect heat resistance by affecting *LOC_Os04g11890.* Likewise, another TE locus associated with leaf survival rate under heat stress was located near a calcium-binding protein gene, *LOC_Os03g07470* (rice annexin *OsANN1*) [50], which could potentially confer enhancement of heat stress tolerance by modulating the production of H_2_O_2_ (Fig. S5f–g, *P* < 0.01). Together, these results indicated that the two dTEs are associated with heat stress.

We also found that a dTE peak harboring a 1.0 kb dTE (*Gypsy*) DEL on chromosome 6 was strongly associated with seed setting rate under cold stress, and this locus was not identified based on SNP data (Fig. 4h; Fig. S5h). This dTE DEL was located 1.6 kb upstream of the bolA family gene *LOC_Os06g28970* [51], which has been linked to stress response pathways. The absence or overexpression of bolA proteins could modulate the response to a variety of environmental challenges [51]. A follow-up analysis showed that the seed setting rate in accessions with the dTE DEL was significantly higher than that in accessions without the dTE DEL (Fig. 4i, *P* < 0.01). The expression level of *LOC_Os06g28970* in accessions with the dTE DEL was significantly higher than that in accessions without the dTE DEL (Fig. 4j, *P* < 0.05). To identify the high-confidence variants predicted to be associated with seed setting rate under cold stress, we performed sum of single effects (SuSiE) [52] analysis to fine-map the dominance loci (PIP = 1) using dTE and SNP variants located near the peak (100 kb on either side). Then, combining these fine-mapping results with the significant TE-GWAS and SNP-GWAS peaks, we confirmed that the 1.0 kb dTE DEL associated with *LOC_Os06g28970* [51] was most likely to be the causal variant affecting seed setting rate under cold stress. These results allowed us to infer that dTE might make widespread contributions to rice complex traits.

## DISCUSSION

Rice is a major food crop for billions of people globally [17], and obtaining a deeper understanding of rice phenotypic variation and crop domestication can significantly benefit agriculture, world food security, and the biological and genomic research communities. In the present study, we took advantage of 15 existing and 232 new high-quality chromosomal-level assemblies of diverse accessions to generate an Asian rice pan-TE map. Subsequently, using three non-Asian rice accessions as outgroups, we used the pan-TE map to infer TE variations representing the derived state, and analyzed their characteristics in Asian rice accessions. Compared to SNPs, TE variations often explain more phenotypic variance and can be used for genomic prediction [53]. The reason for this could be that, as compared with SNPs, TE variations are more frequently the causal mutation. For example, by performing dTE-eQTL analysis, we identified 1246 genes whose expression was significantly associated with dTEs but not identified based on SNP data. Additionally, using dTE data to conduct a GWAS of agronomic traits in rice, we successfully uncovered multiple novel associations that could not be detected using SNP data (Fig. 4h).

Given that knowing the ancestral or derived state of each TE can support inferences about the evolutionary relationships between rice accessions and the genetic basis of phenotypic diversity in the rice populations, these rich dTE data sets represent important resources for investigations of rice evolution and domestication history in functional genomics studies. Using the pan-TE map, we found that the number of dTE INS events was greater than dTE DEL events. TE variation frequency is commonly used to mirror TE age, because low-frequency TE variations are much younger than those in the outgroup [54]. Consistent with this assumption, the vast majority of low-frequency dTE INSs were younger and more quickly removed than dTE DELs in nature, implying that these sequences might be deleterious and selected during artificial domestication or natural selection [55].

The construction of a species’ pan-genome enables us to resolve hidden genomic complexity involving TE variations, and re-evaluate their role in genome evolution and generation of phenotypic variation. Previous studies utilized Asian cultivated rice, 208 *Os* accessions [22], 3010 *Os* accessions [11] and 176 *Os* accessions [56], based on the short-read data, to identify TE and develop a TE data set, without *Or* accessions, while our pan-TE variation map included 218 *Os* and 29 *Or* accessions. We could analyze the changes in TE variation during rice domestication processes, and found that 3935 selected dTEs seem likely to be involved in the domestication process from *Or* to *Osi* or *Osj*. In addition, our use of high-quality chromosome-level genome assemblies enabled us to identify complex TE variation. We found dTEs that contain two or more TEs in our pan-TE map, such as a dTE consisting of one *Gypsy*, one *Helitron* and one *Copia* element, which are also likely to contribute to phenotypic diversity. Using the high-quality TE data set, we could identify TE contributions to environmental adaptation and domestication, and some rare TE mutations may have greater potential for gene cloning and molecular breeding [57].

In addition, our reference-quality genomes will be helpful to the very large number of research groups doing various basic biological studies [58], such as those developing genome editing technologies and promoting successful mapping of causal mutations positioned within highly complex and/or duplicated regions. The Asian pan-TE map also offers a platform that enables researchers to obtain more information than they would using only a few accessions.

## MATERIALS AND METHODS

Detailed materials and methods are available in the supplementary data.

## Supplementary Material

nwae188_Supplemental_Files

## Data Availability

All new assemblies of 232 accessions in the present study have been deposited at the Genome Warehouse (https://bigd.big.ac.cn/gwh/) under PRJCA018476. The TE annotation of all assemblies has been deposited at Figshare (https://figshare.com/articles/dataset/Annotations_of_250_high_quality_rice_genomes/23803956).
