# Supplementary material for: A pan-TE map highlights transposable elements underlying domestication and agronomic traits in Asian rice
Source: Natl Sci Rev. 2024 Jun 4;11(6):nwae188. doi: 10.1093/nsr/nwae188 (PMC11221428; doi:10.1093/nsr/nwae188)
Supplement: nwae188_Supplemental_Files [file nwae188_supplemental_files.zip › Supplemental Figures.pdf]

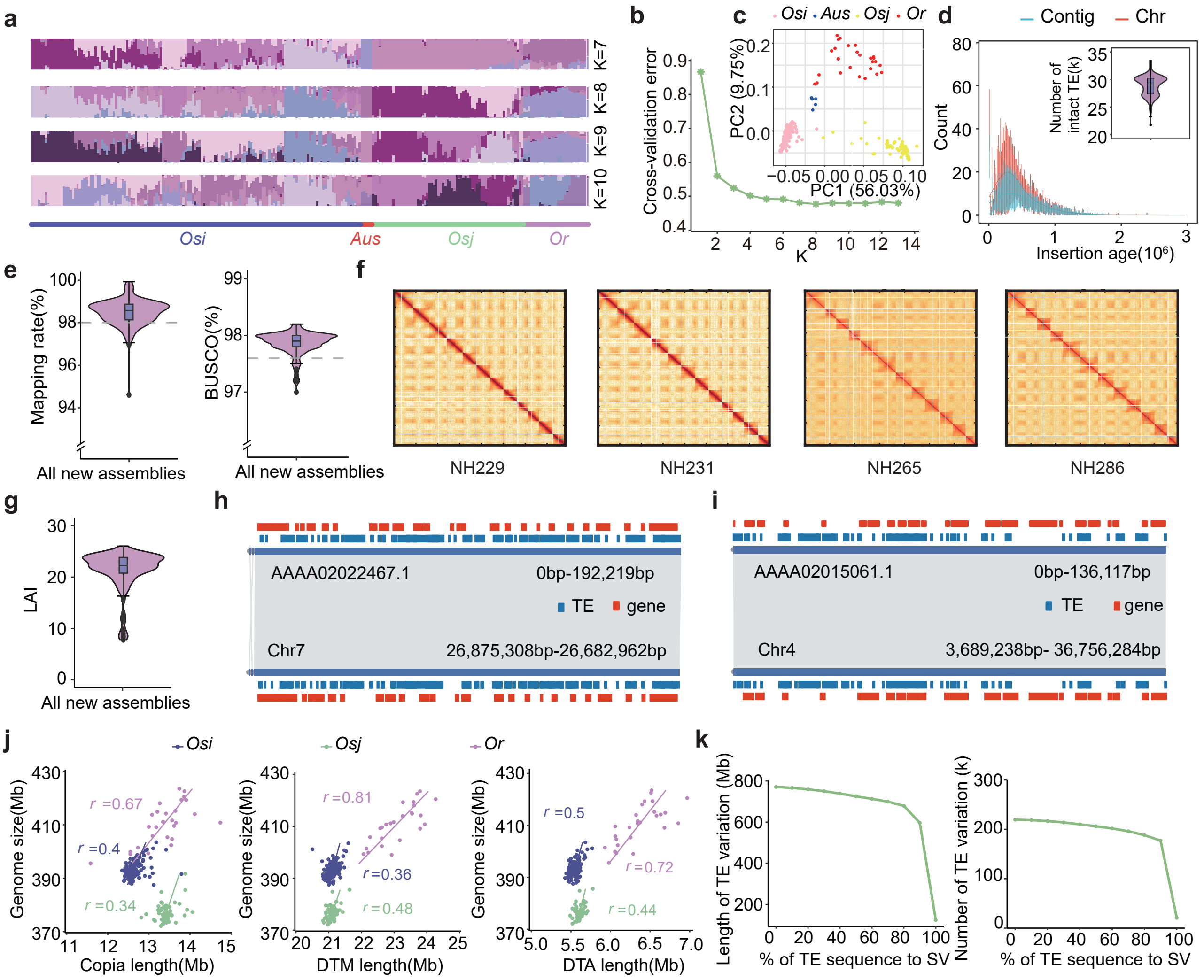

Fig. S1 Population structure, assembly assessment and characteristics of TE variations in Asian rice accessions.

a Population structure of 250 rice accessions inferred by ADMIXTURE. The length of each segment in each vertical bar represents the proportion contributed by ancestral populations (K=7–10). **b** Cross-validation error for K = 1–12 in the ADMIXTURE analysis. When K = 8, the cross validation error was the lowest. **c** Principal component analysis (PCA) of Asian rice accessions, points are colored according to the admixture result. **d** Intact LTR insertion age distribution for chromosomal-level genomes and contig-level genome, and number of intact TEs of the new assemblies across 232 accessions. **e** Mapping rate (**left**) of Illumina short-read data and BUSCO evaluation (**right**) of the new assemblies across 232 accessions. The dashed lines indicate Nipponbare values. **f** Hi-C contact maps for NH229, NH231, NH265 and NH286 with a 100 kb sliding window. **g** Long terminal repeat (LTR) assembly index (LAI) of the new assemblies across 232 accessions. **h-i** Comparison of sequence assembly (**bottom**) with Bacterial Artificial Chromosome (BAC, **top**) clones sequenced using Sanger technology. Genes and TEs are indicated by blue and red boxes, respectively. **j** Pearson correlation coefficients for comparisons between total length of TE families and genome size across different subpopulations. Colored dots and lines indicate data from each subpopulation. **k** Total length (**left**) and number (**right**) of non-redundant TE sequences detected in the Asian accessions. The x axis is the ratio of the length of TE sequences to present/absent variations (PAV) sequences.

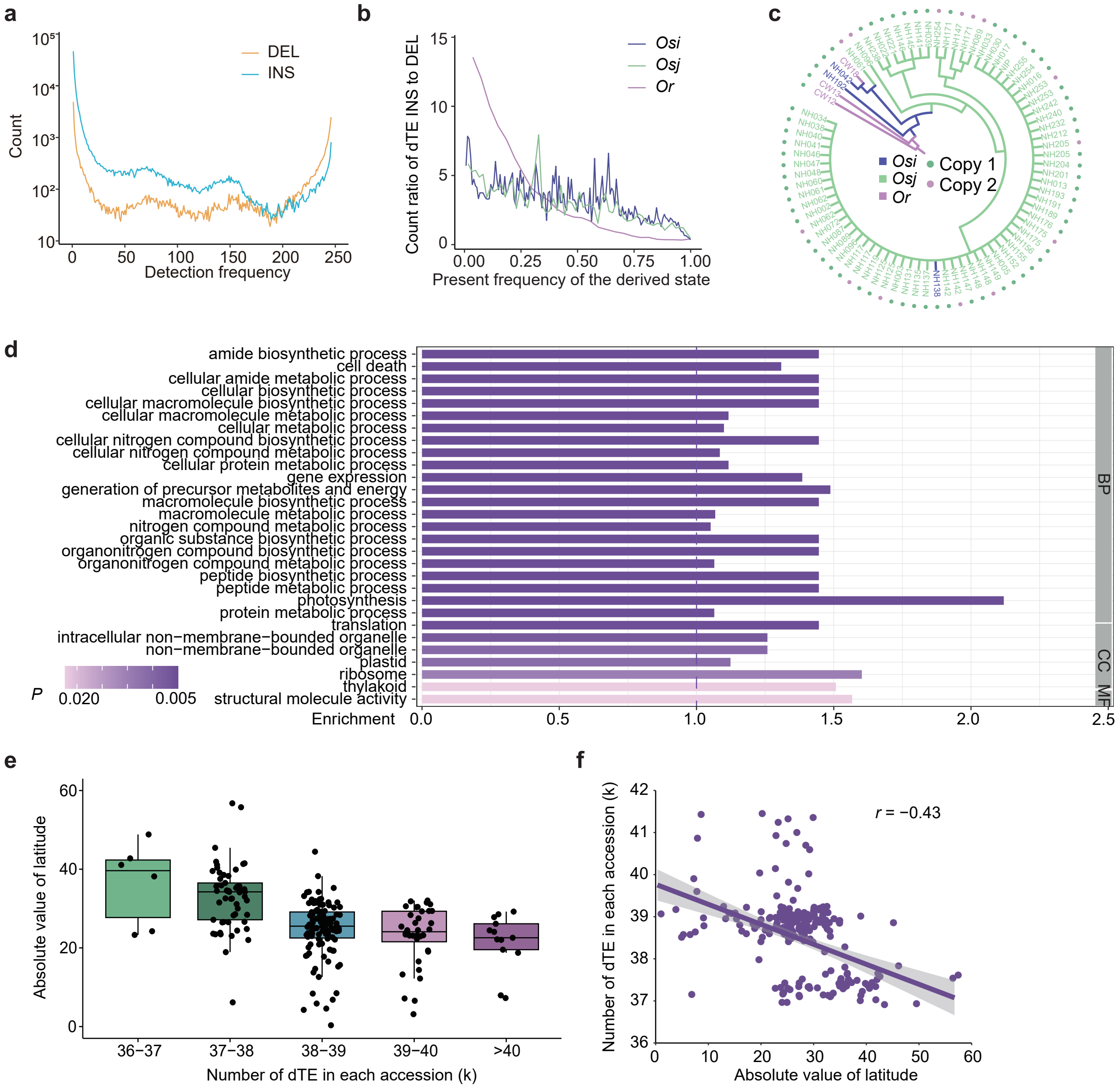

Fig. S2 Characterization of dTEs representing a derived state across Asian accessions.
a Frequencies of derived state for each dTE in 247 Asian accessions. The detection frequency of dTE insertions (INSs) was indicated in orange and that of dTE deletions (DELs) was indicated in blue. **b** The ratio of INS events to DEL events for different frequencies of dTEs in different subpopulations. X axis represents the frequencies of derived state across different subpopulations. **c** Phylogenetic tree of *Helitron* sequences in the promoter of *MYB61* from Asian accessions. Accessions in different subpopulations are indicated by different colors. Different *Helitron* copy numbers are indicated by dots with different colors. **d** Gene ontology analyses of the genes without dTE in their genic regions (within ±2 kb of gene body). **e** The absolute value of latitude of the accessions examined in this study, the x axis indicate the number of dTEs in each accession. **f** Pearson correlation coefficients for comparisons between latitude and the number of dTEs in each accession.

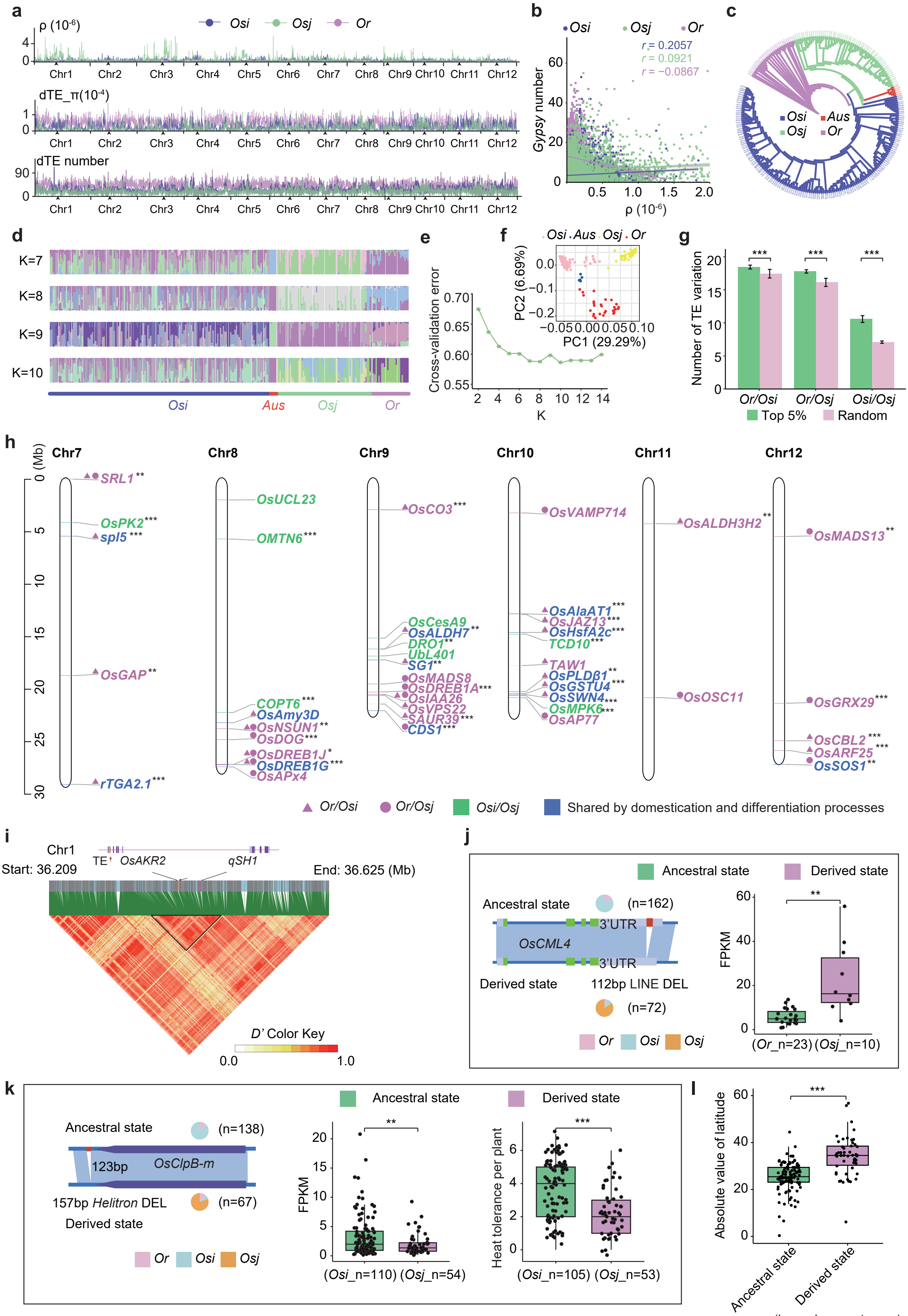

(legend on next page)

Fig. S3 Contribution of dTEs to rice domestication and differentiation.

a Chromosomal distributions of recombination rate (ρ) and dTE diversity values, and numbers of dTEs across different subpopulations. Values were calculated in a 100kb sliding window. The black triangle indicates centromere. **b** Pearson correlation coefficient between *Gypsy* density and recombination rate (ρ) across different subpopulations. **c** Phylogeny of 247 accessions based on pan-TE genotypes. Accessions in different subpopulations are indicated by different colors. **d** Population structure of 247 rice accessions inferred by ADMIXTURE using dTE. The length of each segment in each vertical bar represents the proportion contributed by ancestral populations (K = 7-10). **e** Cross-validation errors for K = 1–14 showing K = 10 had the lowest error. **f** PCA of Asian accessions; points are colored according to the admixture result. **g** Comparisons of the average number of dTEs in the selective windows (i.e., ranked in top 5% of 100 kb F_{ST} windows for SNPs) and in windows between subpopulations, 27 *Osi*, 27 *Osj* and 27 *Or* were randomly selected 500 times from 247 Asian rice. **h** Positions of functional genes harboring selected dTEs in their genic regions on chromosomes 7–12. Genic regions included the gene body, regions of 2kb upstream (promoter) and 2kb downstream (downstream) of the gene body. Functional genes harboring selected dTEs in rice domestication and differentiation processes are displayed in purple and green font, respectively. The shared genes harboring selected dTEs between domestication and differentiation processes are displayed in blue font. Differences in the gene expression between subpopulations were tested using the Student's *t*-test. ****P* < 0.001, ***P* < 0.01, and * *P* < 0.05. **i** Linkage disequilibrium heatmap of the magnified region, which includes a known gene *OsAKR2* harboring a dTE, and a known domestication gene *qSH1*. **j** A deletion (DEL) of a dTE occurred in the 3'UTR of *OsCML4* in upland *Osj* accessions (derived state) but not in *Or* accessions (ancestral state). The expression level of *OsCML4* in upland *Osj* accessions with this dTE DEL was higher than in *Or* accessions without with this dTE DEL. Significance was determined using the Student's *t*-test, ***P* < 0.01. **k** A 157 bp *Helitron* DEL occurred at 123 bp downstream of *OsClpB*-m in *Osj* accessions based on our dTE inference (**left**). Differences in the expression level of *OsClpB-m* (**middle**) and heat tolerance (**right**) between *Osj* accessions with this dTE DEL (derived state) and *Osi* accessions without this dTE DEL (ancestral state). Significance was determined using the Student's *t*-test, ****P* < 0.001, ***P* < 0.01. **l** Differences in the absolute value of latitude of the accessions with this dTE DEL (derived state) and those without this dTE DEL (ancestral state) near *OsClpB-m*.

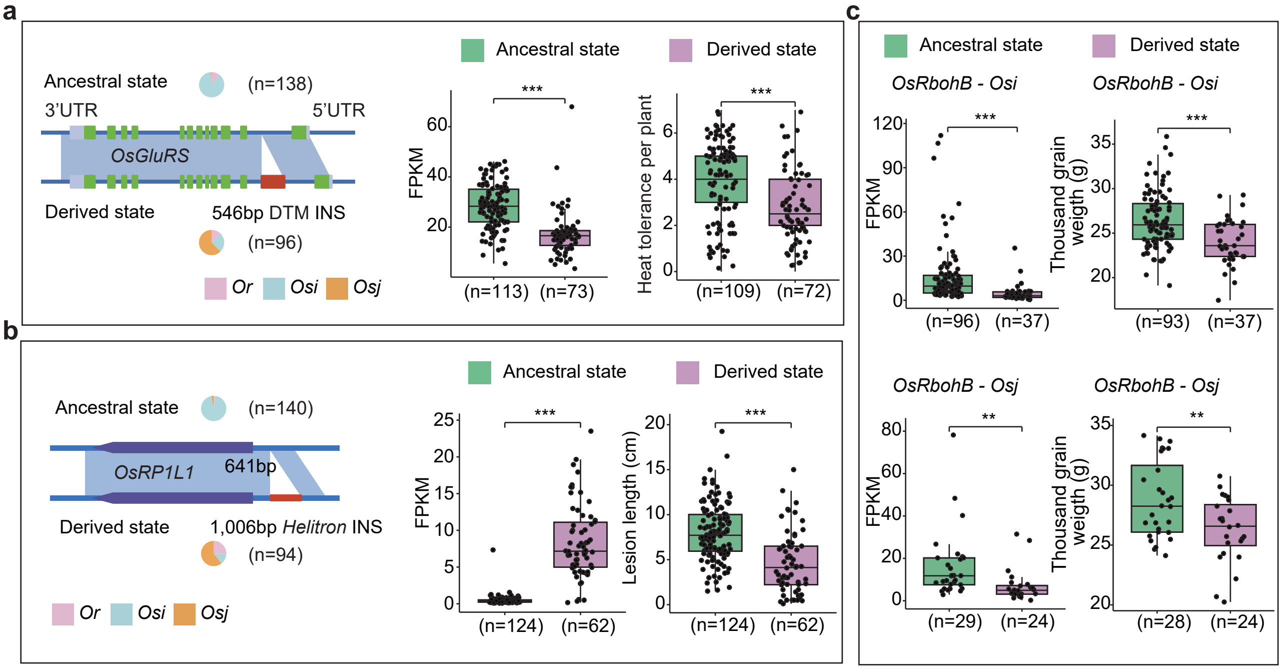

Fig. S4 TE variations associated with gene expression.
a A dTE insertion (INS) occurred in the first intron of a known heat resistance gene, *OsGluRS* (**left**). Differences in expression level of *OsGluRS* (**middle**) and heat resistance (**right**) between accessions with and without the dTE INS. Significance was determined using the Student's *t*-test, ***P < 0.001. **b** A 1.0 kb dTE INS occurred in promoter of a known bacterial blight resistance gene, *OsRP1L1* (**left**). Differences in the expression level of *OsRP1L1* (**middle**) and the lesion length (cm) caused by bacterial blight (Philippines Race P2, **right**) between accessions with and without this dTE INS. Significance was determined using the Student's *t*-test, ***P < 0.001. **c** Comparison of the expression level of *OsRbohB* and thousand grain weight between accessions with and without the dTE INS event in *Osi* subpopulation and *Osj* subpopulation, respectively. Significance was determined using the Student's *t*-test, ***P < 0.001, **P < 0.01.

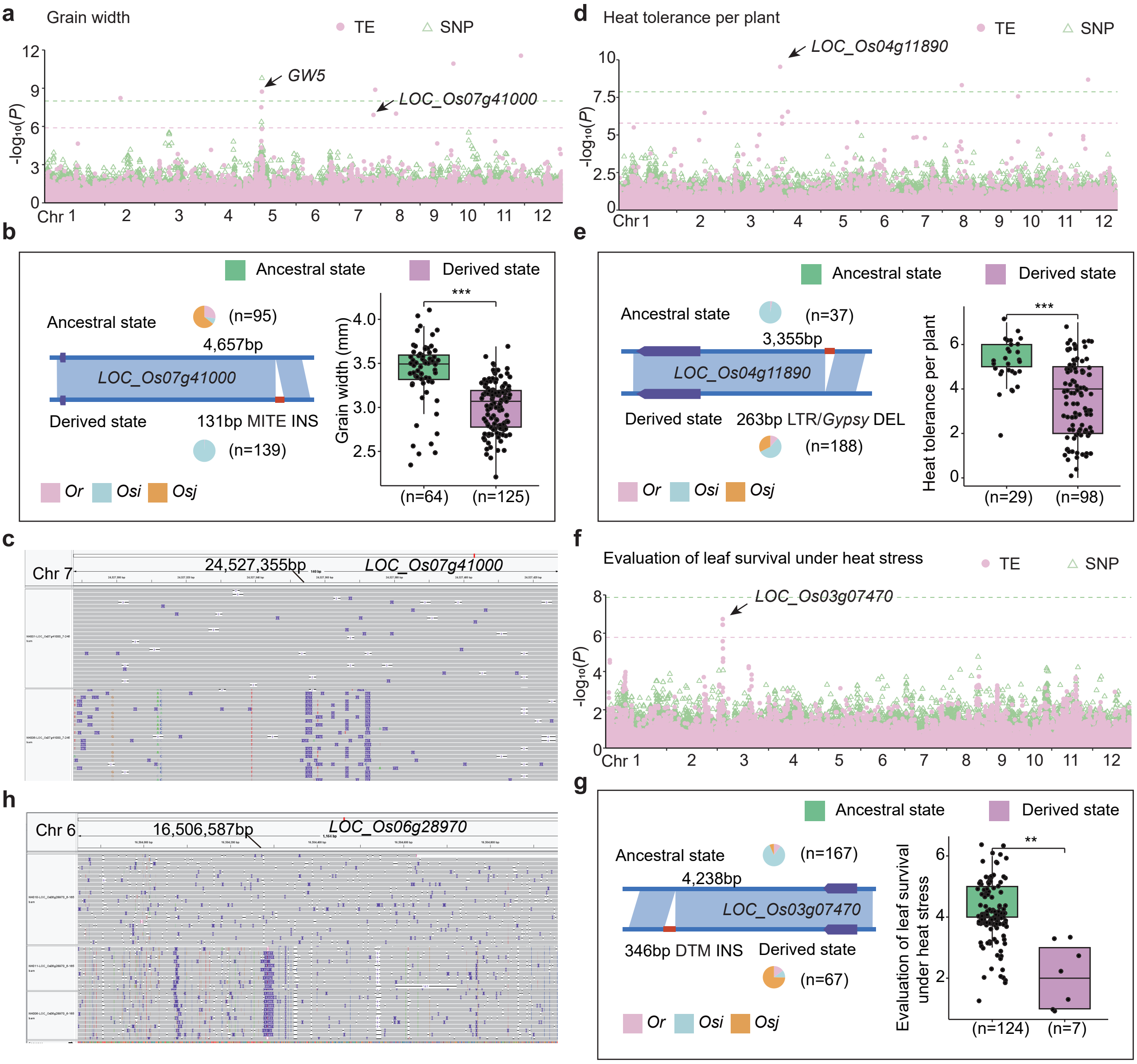

Fig. S5 TE variations associated with agricultural traits.

a GWAS for grain width using dTEs (pink dots) and SNPs (green triangles) dataset for *O. sativa* (*Os*) accessions. In addition to *GW5*, which was previously reported, a locus on chromosome 7 was significantly associated with grain width; this locus was identified using dTEs but not SNP data. **b** The most strongly associated dTE was a *Stowaway* MITE insertion (INS) positioned near (~4.7kb) *LOC_Os07g41000*. The grain width in *Os* accessions with and without the dTE INS were significantly different. Significance was determined using the Student's *t*-test, ***$P < 0.001$. **c** Breakpoints of the INS near the *LOC_Os07g41000* gene in *Os* were validated by mapping ONT reads to the Nipponbare reference genome. **d** GWAS for heat tolerance per plant using dTEs (pink dots) and SNPs (green triangles) of *Osi* accessions. A dTE (263bp *Gypsy*) deletion (DEL) near *LOC_Os04g11890* was detected using dTEs but not SNP data. **e** The difference in heat tolerance between *Osi* accessions with and without the dTE DEL. Significance was determined using the Student's *t*-test, ***$P < 0.001$. **f** GWAS for evaluation of leaf survival under heat stress using dTEs (pink dots) and SNPs (green triangles) of *Osi* accessions. A dTE (346bp DTM) INS near *LOC_Os03g07470* was detected using dTEs but not SNP data. **g** Leaf survival rate under heat stress was significantly different between *Osi* accessions with and without the dTE. Significance was determined using by the Student's *t*-test, **$P < 0.01$. **h** Breakpoints of the 1.0 kb INS (i.e., DEL against outgroup gnome) in the *LOC_Os06g28970* promoter region in *Os* were validated by mapping ONT reads to the Nipponbare reference genome.

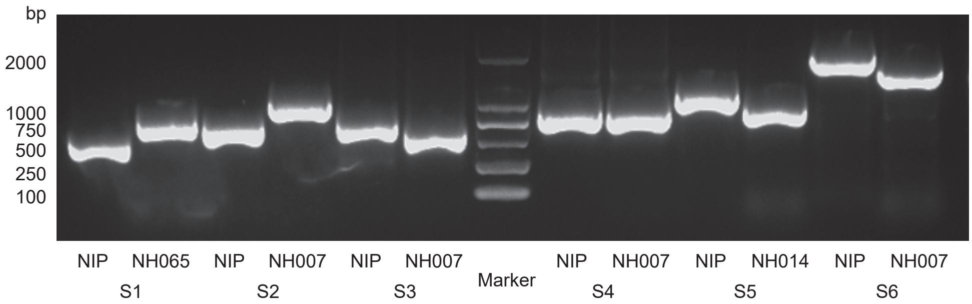

Fig. S6 The PCR validation of the randomly selected TE variations.
