# Supplementary material for: A pan-TE map highlights transposable elements underlying domestication and agronomic traits in Asian rice
Source: Natl Sci Rev. 2024 Jun 4;11(6):nwae188. doi: 10.1093/nsr/nwae188 (PMC11221428; doi:10.1093/nsr/nwae188)
Supplement: nwae188_Supplemental_Files [file nwae188_supplemental_files.zip › Supplemental Materials and Methods.pdf]

Supplementary information for

A pan-TE map highlights transposable elements underlying domestication and agronomic traits in Asian rice

Xiaoxia Li[1,2#], Xiaofan Dai[1,2#], Huiying He[1#], Yang Lv[1,2#], Longbo Yang[1#], Wenchuang He[1], Congcong Liu[1], Hua Wei[1], Xiangpei Liu[1], Qiaoling Yuan[1], Xianmeng Wang[1], Tianyi Wang[1], Bintao Zhang[1], Hong Zhang[1], Wu Chen[1], Yue Leng[1], Xiaoman Yu[1], Hongge Qian[1], Bin Zhang[1], Mingliang Guo[1], Zhipeng Zhang[1], Chuanlin Shi[1], Qianqian Zhang[1], Yan Cui[1], Qiang Xu[1], Xinglan Cao[1], Dandan Chen[1], Yongfeng Zhou[1,4]*, Qian Qian[1,3,5]*, Lianguang Shang[1,5]*

ADDRESS

[1]Shenzhen Branch, Guangdong Laboratory of Lingnan Modern Agriculture, Genome Analysis Laboratory of the Ministry of Agriculture and Rural Affairs, Agricultural Genomics Institute at Shenzhen, Chinese Academy of Agricultural Sciences, Shenzhen, 518120, China

[2]Rice Research Institute, Shenyang Agricultural University, Shenyang 110866, China

[3]State Key Laboratory of Rice Biology, China National Rice Research Institute, Hangzhou, 310006, China

[4]National Key Laboratory of Tropical Crop Breeding, Tropical Crops Genetic Resources Institute, Chinese Academy of Tropical Agricultural Sciences, Haikou, 571101 , China

[5]Yazhouwan National Laboratory, No. 8 Huanjin Road, Yazhou District, Sanya City, Hainan Province, 572024, China

[#] These authors contributed equally to this work.

*Correspondence: shanglianguang@caas.cn, qianqian188@hotmail.com, zhouyongfeng@caas.cn.

This PDF file include:

Supplemental materials and methods

Figures S1 to S5

REFERENCES

Supplemental materials and methods

Materials and data collection

We downloaded relevant ONT long reads of 234 rice accessions from public databases, of these one *O. glaberrima*, one *O. barthii*, 202 *Os* and 28 *Or* accessions were downloaded from NCBI under PRJNA656318, two *Os* accessions were downloaded from Genome Sequence Archive (GSA) in the National Genomics Data Center under accession number CRA003081. We also downloaded relevant PacBio long reads of 16 rice accessions from public databases, including one *Or* accession (from GSA under PRJCA002385) and 15 *Os* accessions (from NCBI under PRJNA565484, PRJNA424001, PRJNA564615, PRJNA565479, PRJNA563359, PRJNA564572, PRJNA565481, PRJNA564763, PRJNA577228, PRJNA565480, PRJNA565483, PRJNA48429, SRR13280200 and SRX6716809; from GSA under project PRJCA000313). Moreover, the Illumina short reads of 250 accessions were from public databases (from NCBI under PRJNA692836, PRJNA318714, SRP226078, SRP226079, SRP226080, SRP226082, SRP226084, SRP226085, SRP226086, SRP226087, SRP226088, SRP226093, SRP227255, SRR13288213, SRR13285939; from GSA under accession number CRA003081 and PRJCA002385), including one *O. glaberrima*, one *O. barthii*, 218 *Os* and 29 *Or* accessions. 18 chromosome-level assemblies were collected from public databases, including one *Or* accession (from GSA under PRJCA002385) and 17 *Os* accessions (from NCBI under PRJNA565484, PRJNA424001, PRJNA564615, PRJNA565479, PRJNA563359, PRJNA564572, PRJNA565481, PRJNA564763, PRJNA577228, PRJNA565480, PRJNA565483, PRJNA318714, PRJNA48429, PRJNA302542 and PRJNA302543; from Genome Warehouse in National Genomics Data Center under accession numbers GWHAORT00000000 and GWHAORU00000000). Leaf transcriptome data of 202 *Os* were from our previous study [1-5] in NCBI website under PRJNA692672. Meanwhile, Hi-C sequencing data of four accessions (NH229, NH231, NH265, and NH286) were collected from NCBI with PRJNA693366. Detailed information of these accessions was provided in Supplementary **Table S1a**.

SNP calling

Raw Illumina short reads from 250 rice gDNA samples were trimmed with Trimmomatic (parameters ‘ILLUMINACLIP:2:30:10 MINLEN:75 LEADING:20 TRAILING:20

SLIDINGWINDOW:5:20', version 0.36) [6]. Subsequently, clean reads were mapped to the NIP reference genome (MSUv7) [7] using Burrows-Wheeler Aligner (BWA, version 0.7.17-r1188) [8] with default parameters, and BCF files were generated using SAMtools (mpileup, version 1.8) [9]. Then BCFtools (version 1.8) [9] was used for SNP calling and filtering (DP < 3 and quality score < 30). SNP calls were further filtered based on the following parameters: (i) integrity ≥ 80% (ii) consensus quality ≥ 40; (iii) site is diallelic and (iv) minor allele frequencies ≥ 0.05.

Phylogenetic tree and population structure

The maximum likelihood phylogenetic tree based on SNPs was built using FastTree (version 2.1.11) [10] with the Jones-Taylor-Thornton CAT model and 20 rate categories. iTOL (version 6.3.1) [11] was used for a tree visualization. Likewise, the maximum likelihood phylogenetic tree based on TE dataset of Asian accessions (See "**Identification and validation of TE**" in Methods), sequences of TE and genes (such as *LIP19*), and sequences of a TE were conducted in FastTree.

For SNP and TEs variation data of 250 accessions, the population structure inference were conducted using PLINK (version v1.90b6.26) [12] and ADMIXTURE (version 1.3.0) [13] with default parameters. PCA based on SNP and TE variations respectively were performed using PLINK (version v1.90b6.26) [12] with parameters '--pca 10'.

De novo genome assembly and assessment

Due to the fact that the assemblies of 232 accessions are not at the chromosome-level [1], we de novo assembled these rice accessions with a pipeline as following steps: (I) the raw Nanopore long reads with quality less than 7 were filtered, the remaining long reads were assembled using NextDenovo [14] (version 2.4, parameters: genome size = 390 Mb, read cutoff = 1k, and seed depth = 45). And raw contigs were polished three times with Nanopore clean long reads and Illumina short reads using NextPolish [15] (version 1.4.1) with default parameters; (II) to further improve single base accuracy, Illumina short reads were aligned to their assembly with BWA (version 0.7.17-r1188) [8], and the mutation sites in each accession assembly were identified with FreeBayes (version 1.3.1) [16]. Variants were filtered with parameters 'QUAL > 20 and DP > 10 and AO > 10' and the homozygous sites were replaced. Then purge Haplotigs (version 1.0.3) [17] was applied to each assembly with low, middle, and high read depth cutoff tuned artificially to

remove false duplications from assemblies. Then the various contaminating DNA from archaea, bacteria, viruses, fungi, and other metazoans were aligned to the NCBI Nr database (downloaded on 4 June 2021) using DIAMOND (version 0.9.24) [18] with parameters '-evalue 1e-5', and contigs in which more than 50% of the sequences of protein-coding annotations aligned to non-viridiplantae organisms were considered contaminants and filtered out; (III) all super contigs of four accessions (NH229, NH231, NH265 and NH286) were achieved to chromosome-level scaffolds using Hi-C pair reads with HiC-Pro [19] (version 3.1.0, default parameters) at 100 kb resolution. And the interaction maps were visualized with HiCPlotter [20] (Supplementary **Fig. S1f**). According to the phylogenetic tree based on whole-genome SNPs (**Fig. 1a**), chromosome-level scaffolds of all super contigs from the remaining 228 accessions were achieved using RaGOO RagTag (version 2.1.0) [21] (Supplementary **Table S1b**), which invoked Minimap2 at the mapping step and MUMmer (version 4.0.0, beta) [22] at the checking step, and found an average of 99.7% ± 0.2% contigs were anchored to the chromosomes eventually.

To evaluate the quality of each genome assembly, completeness of assemblies was firstly evaluated through alignment to the NIP reference genome [7] by MUMmer [22] (version 4.0.0, parameters: '--mum -t 10 -c 90 -l 40'), and further manually modified when there was a directional error in the assembly sequence. The average of 99.9996% ± 0.0004% high accuracy at the single-base level (Supplementary **Table S1b**) based on the count of homozygous SNPs, and this value was well below one error per 10,000 bases, a quality standard used for high-quality genomes such as Nipponbare [7] genome. BUSCO [23] evaluation of each assembly showed an average of 97.8% ± 0.2% of the 1,440 single copy Embryophyta genes (Supplementary **Table S1b**), and the BUSCO of NIP genome [7] was 97.6% using the same way. Moreover, to estimate mapping rates, Illumina short reads were mapped to each final assembly and found the average completeness of each assembly was 98.5% ± 0.5% (Supplementary **Table S1b**), which highlights the completeness of the new assemblies [1].

Repeat sequence annotation and assessment

Extensive de novo TE Annotator (EDTA, version 1.9.6) [24] was used to annotate repeat sequences of the 250 rice assemblies using its curated TE library (rice 6.9.5.liban) with parameters '--overwrite 1 --sensitive 1 --anno 1' (Supplementary **Table S1c**), which combined the raw

predictions of LTR_Finder (version 1.07) [25], LTRharvest (version 1.5.10) [26], LTR_retriever (version 2.9.0) [27], Generic Repeat Finder (version 1.0) [28], TIR-Learner (version 1.23) [29], RepeatModeler (version 2.0.1), RepeatMasker (www.repeatmasker.org, version 4.1.2-p1) and HelitronScanner (version 1.0) [30] with extra basic and advanced filters.

To evaluate the integrity of the TE sequence in each assembly, LAI of each new assembly was calculated using LTR_retriever (version 2.9.0) [27] with default parameter, and found that the average of LAI for each assembly reached the "gold standard" level (Supplementary **Table S1b** and **Fig. S1g**). In addition, the sequences of 9311 were randomly selected and aligned to the raw BAC sequence of 9311 using Blastn with an e-value of 1e-10. Comparison of sequence assembly with BAC clones sequenced by Sanger technology showed higher quality of assembled sequence coverage in the gene and TEs regions (Supplementary **Table S1d** and **Fig. S1h-i**).

TE identification

To survey the landscape of TE polymorphism in Asian rice, presence/absence variants (PAVs, > 50 bp) were inferred from 250 high-quality assemblies against the Nipponbare [7] reference genome using the whole-genome comparison by minimap2 (version 2.24-r1122) [31] and then processed with CHAIN/NET/NETSYNTENY tools [32]. Calling of presence/absence variants (PAVs, >50 bp) and further filtering were performed using custom perl scripts which are available at https://github.com/yiliao1022/LASTZ_SV_pipeline. PAVs genotype (including heterozygous and homozygous genotypes) were further identified using ONT or PacBio long reads with SVJedi [33] (version 1.1.6). A PAV was regarded as TE variation when more than 90% of the PAV sequence was TE sequence (Supplementary **Fig. S1k**).

Inferring the derived TEs

To infer the ancestral or derived state of TE variations, we used three non-Asian accessions, including one *O. glaberrima*, one *O. barthii* and one *O. glumaepatula* (https://www.ncbi.nlm.nih.gov/nuccore/CM002522.2), as outgroups for an analysis inferring whether a given TE variation was in derived or ancestral state in a given accession. A TE variation, has varied states (i.e., both ancestral state and derived state) among the Asian rice accessions, was defined as derived TE variation (dTE). Ancestral state indicates that the genotype of the TE

variation in a given accession (0/0) is the same as outgroups (0/0), while derived state indicates the genotype of the TE variation in a given accession (1/1 or 0/1) is different from outgroups (0/0), including homozygous (1/1) and heterozygous (0/1) genotype. Finally, a dTE genotype dataset in matrix format is generated for use in downstream analysis, including domestication, gene expression, and GWAS.

TE validation

To quantitatively estimate the accuracy of TE calling, 200 randomly selected TEs were manually examined by visualizing the corresponding long read alignment through an Integrative Genomics Viewer Browser [34]. The TE calling accuracy was estimated to be 97.5% (Supplementary, **Table S1e**). Several typical TE variations from our pan-TE map were validated by PCR (Supplementary **Fig. S6**). Primers were showed in Supplementary **Table S1f**.

The insertion time of TE

The *Helitron* in the *MYB61* promoter region was structurally intact and then the insertion times of the intact *Helitron* family were calculated based on a previously published approach [35, 36]. First, this *Helitron* sequence was captured in each accession of five subpopulations, and a multiple alignment file was generated for the *Helitron* family that contains using MAFFT (version 7.305b) with FFT-NS-2 method [37, 38]. A consensus sequence of the *Helitron* family was extracted from the multiple alignment, and the sequence divergence between each TE copy and the consensus sequence was calculated using EMBOSS (version 6.5.7.0) [39]. The TE insertion time for each *Helitron* copy was estimated based on the sequence divergence, and a substitution rate of 1.3×10^{-8} per site per year was used to calculate insertion times [40-42] (Supplementary **Table S1g**).

Domestication and differentiation

The relative divergence measure F_{ST} was estimated to identify the divergent regions during domestication and differentiation regions between subpopulations. F_{ST} was calculated using VCFtools (version 0.1.16) [43] for each TE variations and the values were ranked, and TE variations with the top 5% of values were selected as highly divergent regions.

In order to identify whether the distribution of TE dataset on the genome was biased, we calculated the number of TE in selective windows (the top 5% 100kb sliding windows with F_{ST}

value) based on the whole-genome SNPs from a previous report [1], including those for *Or-Osi*, *Or-Osj* and *Osi-Osj* comparisons. Meanwhile, 500 independent permutations of selective windows were performed to evaluate whether TEs were enriched in the real selective regions than expected at random.

eQTL analysis

TE variants of 187 *Os* accessions with parameters '--maf 0.05 --max-missing 0.8' were filtered using VCFtools [43]. PCA was conducted to infer population structure using Plink2 [44] (version 2.00a3LM, parameters: -pca 10). Both first 10 principal components in PCA and 20 factors in probabilistic estimation of expression residuals (PEER, from our previous study) [1] results were used as covariates. The linear regression model of the MatrixEQTL package (version 2.2) [45] was used to detect associations between TE-gene pairs (the gene expression data and the results of SNP-eQTL were from our previous study) [1]. *P*-value = 5.314e-11 (0.05/TE markers 23,659) was used as the threshold.

GWAS analysis

GWAS was conducted using two different datasets, including TE and SNP variants datasets. On one hand, TEs markers from our TE dataset were filtered by VCFtools (version 0.1.13) [43] with parameters '--maf 0.05 --max-missing 0.8'. Phenotypes grain width, heat stress and seed setting rate under cold stress in the present study were acquired from the work of Shang et al. [1], Yang et al [46]. and Zhang et al [47]., respectively. PCA was conducted by Plink2 [44] (version 2.00a3LM, parameters: -pca 10). First five principal components and standardized GEMMA matrix of kinship (version 0.98.1 -gk 2) were used as covariates. GWAS was performed using a mixed linear model in genome-wide efficient mixed model in GEMMA (version gemma-0.98.5, parameters: -lmm 4 -k) in *Os* and *Osi* subpopulations, respectively. The threshold for GWAS was calculated using Bonferroni correction (0.05/TE markers). Manhattan plot and QQ plot were created using the CMplot [48] R-package.

Linkage disequilibrium analysis

We measured the LD of the region (36.209Mb to 36.625 Mb of chromosome 1) containing a known domestication gene *qSh1*, a known gene *OsAKR2* and a divergent dTE INS near *OsAKR2*.

The dataset consisting of SNP and TE variations was filtered using VCFtools [43] (--maf 0.05 --max-missing 0.8) in Asian rice accessions, and then were used to perform the LD analysis using LDBlockShow (version 1.35) [49].

To measure the LD between TEs and nearby SNPs/InDels, we first used VCFtools [43] to filter SNP and InDels (--maf 0.05 --max-missing 0.8), and TEs (--maf 0.05 --max-missing 0.8) of Asian rice accessions. Then, for each TE variation, the maximum R^2 with adjacent SNPs and InDels within 50 kb on either side is calculated by Plink2 [44] with parameters: --r2 --ld-window-r2 0 --ld-window-kb 50000.

Estimation of recombination rate

Genome-wide population recombination rates were estimated in each subpopulation using SNP dataset by pyrho [50, 51] (version 0.1.0, default parameters). We first used SNP data to infer population size histories for three subpopulations (*Osi*, *Osj* and *Or* subpopulations) by smc++. Since pyrho [50, 51] (version 0.1.0) has a command line interface and consists of a number of separate commands, a typical workflow is to first use make_table of pyrho [50, 51] to build a lookup table for the results of smc++ (version 1.15.2, https://github.com/popgenmethods/smcpp), and then use hyperparam to find reasonable hyperparameter settings for the results of make_table. Finally, using optimize of pyrho [50, 51] to infer a fine-scale recombination map with default parameters based on the results of hyperparameter and SNP dataset across different subpopulations. The recombination map was calculated per 100 kb sliding window by perl script across different subpopulations.

Bacterial blight treatment and phenotyping

To investigate the impact on rice bacterial blight resistance, artificial inoculation experiments were conducted on a population of 206 Asian cultivated rice. Briefly, adult seedlings of each rice accession were grown in the field at the Shunyi experimental station of the Institute of Crop Sciences (40.23 N, 116.57 E) in Beijing from June to October 2021. The P2 [PXO86] strain from the Philippines was used for inoculation with leaf clipping methods. Lesion length (cm) was measured 15 days after inoculation as an indicator of resistance.

Cold stress treatment and phenotyping

Seeds were immersed in distilled water for three days until germination and then grown in seed trays for ten days under the control conditions (16 h light at 30°C and 8 h darkness at 28°C), including NH181 (an *Osi* accession) and NH242 (an *Osj* accession) accessions. The seedlings were then subjected to cold-stress treatment at 4°C for 72h, and were then returned to the control conditions for recovery. For phenotyping, survival scores were recorded after a four-day recovery period from the cold-stress treatment. Numerical survival scores ranging from 1 to 5 were assigned based on evaluation of whole plant morphology. For cold stress, a score of 1 indicates the lowest tolerance and a score of 5 indicates the highest tolerance. More than 30 seedlings per accession were used in the cold-stress treatments. Experiments were performed with at least three biological replicates for each of the seedlings.

GO enrichment analysis

GO enrichment analysis was applied to analyze the biological significance, which includes biological processes (BP), cellular components (CC) and molecular functions (MF) [52]. The GO background comment and annotation information were downloaded from the GENEONTOLOGY website (http://geneontology.org/docs/download-ontology/) and eggNOG website [53] (http://eggnogdb.embl.de/#/app/emapper). GO enrichment analysis was performed using the TBtools [54] (version 1.108). Adjusted $P < 0.05$ was considered as significant. The R package 'ggplot' was used to achieve GO visualization (Supplementary **Table S1h**).

Transient expression assay of promoter activity

To assay the effect of dTE on *OsRbohB* expression, *OsRbohB* promoter fragments were amplified from two rice varieties, NH016 (without dTE INS) and NH243 (with dTE INS), and inserted into the LUC vector. Rice protoplasts were isolated from stems of seedlings grown in the dark for one week. 15-20 μg of plasmid was transformed into rice protoplasts for transient expression. LUC to REN luciferase activities were measured by the dual-luciferase Reporter Assay System (Promega). The primers used are listed in Supplementary **Table S1f**.

REFERENCES

1. Shang L, Li X, He H *et al.* A super pan-genomic landscape of rice. *Cell Res*. 2022; **32**(10): 878-896. doi: 10.1038/s41422-022-00685-z

2. Song JM, Xie WZ, Wang S *et al.* Two gap-free reference genomes and a global view of the centromere architecture in rice. *Mol Plant*. 2021; **14**(10): 1757-1767. doi: 10.1016/j.molp.2021.06.018
3. Qin P, Lu H, Du H *et al.* Pan-genome analysis of 33 genetically diverse rice accessions reveals hidden genomic variations. *Cell*. 2021; **184**(13): 3542-3558 e3516. doi: 10.1016/j.cell.2021.04.046
4. Zhang H, Wang Y, Deng C *et al.* High-quality genome assembly of Huazhan and Tianfeng, the parents of an elite rice hybrid Tian-you-hua-zhan. *Sci China Life Sci*. 2022; **65**(2): 398-411. doi: 10.1007/s11427-020-1940-9
5. Zhou Y, Chebotarov D, Kudrna D *et al.* A platinum standard pan-genome resource that represents the population structure of Asian rice. *Sci Data*. 2020; **7**(1): 113. doi: 10.1038/s41597-020-0438-2
6. Bolger AM, Lohse M, Usadel B. Trimmomatic: a flexible trimmer for Illumina sequence data. *Bioinformatics*. 2014; **30**(15): 2114-2120. doi: 10.1093/bioinformatics/btu170
7. Kawahara Y, de la Bastide M, Hamilton JP *et al.* Improvement of the *Oryza sativa* Nipponbare reference genome using next generation sequence and optical map data. *Rice (N Y)*. 2013; **6**(1): 4. doi: 10.1186/1939-8433-6-4
8. Li H, Durbin R. Fast and accurate short read alignment with Burrows-Wheeler transform. *Bioinformatics*. 2009; **25**(14): 1754-1760. doi: 10.1093/bioinformatics/btp324
9. Danecek P, Bonfield JK, Liddle J *et al.* Twelve years of SAMtools and BCFtools. *Gigascience*. 2021; **10**(2). doi: 10.1093/gigascience/giab008
10. Price MN, Dehal PS, Arkin AP. FastTree: computing large minimum evolution trees with profiles instead of a distance matrix. *Mol Biol Evol*. 2009; **26**(7): 1641-1650. doi: 10.1093/molbev/msp077
11. Letunic I, Bork P. Interactive Tree Of Life (iTOL) v5: an online tool for phylogenetic tree display and annotation. *Nucleic Acids Research*. 2021; **49**(W1): W293-W296. doi: 10.1093/nar/gkab301
12. Purcell S, Neale B, Todd-Brown K *et al.* PLINK: a tool set for whole-genome association and population-based linkage analyses. *Am J Hum Genet*. 2007; **81**(3): 559-575. doi: 10.1086/519795
13. Alexander DH, Novembre J, Lange K. Fast model-based estimation of ancestry in unrelated individuals. *Genome Res*. 2009; **19**(9): 1655-1664. doi: 10.1101/gr.094052.109
14. Hu, J. et al. An efficient error correction and accurate assembly tool for noisy long reads. *bioRxiv* 2023.**03**.09.531669 (2023) doi:10.1101/2023.03.09.531669.
15. Hu J, Fan J, Sun Z *et al.* NextPolish: a fast and efficient genome polishing tool for long-read assembly. *Bioinformatics*. 2020; **36**(7): 2253-2255. doi: 10.1093/bioinformatics/btz891
16. Garrison E, Marth G. Haplotype-based variant detection from short-read sequencing. *arXiv preprint arXiv:12073907*. 2012.

17. Roach MJ, Schmidt SA, Borneman AR. Purge Haplotigs: Allelic contig reassignment for third-gen diploid genome assemblies. *BMC Bioinformatics*. 2018; **19**(1).
18. Buchfink B, Xie C, Huson DH. Fast and sensitive protein alignment using DIAMOND. *Nat Methods*. 2015; **12**(1): 59-60. doi: 10.1038/nmeth.3176
19. Servant N, Varoquaux N, Lajoie BR *et al.* HiC-Pro: an optimized and flexible pipeline for Hi-C data processing. *Genome Biol*. 2015; **16**: 259. doi: 10.1186/s13059-015-0831-x
20. Akdemir KC, Chin L. HiCPlotter integrates genomic data with interaction matrices. *Genome Biol*. 2015; **16**(1): 198. doi: 10.1186/s13059-015-0767-1
21. Alonge M, Lebeigle L, Kirsche M *et al.* Automated assembly scaffolding using RagTag elevates a new tomato system for high-throughput genome editing. *Genome Biol*. 2022; **23**(1): 258. doi: 10.1186/s13059-022-02823-7
22. Marçais G, Delcher AL, Phillippy AM *et al.* MUMmer4: A fast and versatile genome alignment system. *PLoS Comput Biol*. 2018; **14**(1): e1005944. doi: 10.1371/journal.pcbi.1005944
23. Simão FA, Waterhouse RM, Ioannidis P *et al.* BUSCO: assessing genome assembly and annotation completeness with single-copy orthologs. *Bioinformatics*. 2015; **31**(19): 3210-3212. doi: 10.1093/bioinformatics/btv351
24. Ou S, Su W, Liao Y *et al.* Benchmarking transposable element annotation methods for creation of a streamlined, comprehensive pipeline. *Genome Biol*. 2019; **20**(1): 275. doi: 10.1186/s13059-019-1905-y
25. Xu Z, Wang H. LTR_FINDER: an efficient tool for the prediction of full-length LTR retrotransposons. *Nucleic Acids Res*. 2007; **35**(Web Server issue): W265-268. doi: 10.1093/nar/gkm286
26. Ellinghaus D, Kurtz S, Willhoeft U. LTRharvest, an efficient and flexible software for de novo detection of LTR retrotransposons. *BMC Bioinformatics*. 2008; **9**: 18. doi: 10.1186/1471-2105-9-18
27. Ou S, Jiang N. LTR_retriever: A Highly Accurate and Sensitive Program for Identification of Long Terminal Repeat Retrotransposons. *Plant Physiol*. 2018; **176**(2): 1410-1422. doi: 10.1104/pp.17.01310
28. Shi J, Liang C. Generic Repeat Finder: A High-Sensitivity Tool for Genome-Wide De Novo Repeat Detection. *Plant Physiol*. 2019; **180**(4): 1803-1815. doi: 10.1104/pp.19.00386
29. Su W, Gu X, Peterson T. TIR-Learner, a New Ensemble Method for TIR Transposable Element Annotation, Provides Evidence for Abundant New Transposable Elements in the Maize Genome. *Mol Plant*. 2019; **12**(3): 447-460. doi: 10.1016/j.molp.2019.02.008
30. Xiong W, He L, Lai J *et al.* HelitronScanner uncovers a large overlooked cache of *Helitron* transposons in many plant genomes. *Proc Natl Acad Sci U S A*. 2014; **111**(28): 10263-10268. doi: 10.1073/pnas.1410068111
31. Li H. Minimap2: pairwise alignment for nucleotide sequences. *Bioinformatics*. 2018; **34**(18):

3094-3100. doi: 10.1093/bioinformatics/bty191

32. Kent WJ, Baertsch R, Hinrichs A *et al.* Evolution's cauldron: duplication, deletion, and rearrangement in the mouse and human genomes. *Proc Natl Acad Sci U S A*. 2003; **100**(20): 11484-11489. doi: 10.1073/pnas.1932072100
33. Lecompte L, Peterlongo P, Lavenier D *et al.* SVJedi: genotyping structural variations with long reads. *Bioinformatics*. 2020; **36**(17): 4568-4575. doi: 10.1093/bioinformatics/btaa527
34. Thorvaldsdóttir H, Robinson JT, Mesirov JP. Integrative Genomics Viewer (IGV): high-performance genomics data visualization and exploration. *Brief Bioinform*. 2013; **14**(2): 178-192. doi: 10.1093/bib/bbs017
35. SanMiguel P, Gaut BS, Tikhonov A *et al.* The paleontology of intergene retrotransposons of maize. *Nat Genet*. 1998; **20**(1): 43-45. doi: 10.1038/1695
36. Kou Y, Liao Y, Toivainen T *et al.* Evolutionary Genomics of Structural Variation in Asian Rice (*Oryza sativa*) Domestication. *Mol Biol Evol*. 2020; **37**(12): 3507-3524. doi: 10.1093/molbev/msaa185
37. Katoh K, Misawa K, Kuma K *et al.* MAFFT: a novel method for rapid multiple sequence alignment based on fast Fourier transform. *Nucleic Acids Res*. 2002; **30**(14): 3059-3066. doi: 10.1093/nar/gkf436
38. Katoh K, Standley DM. MAFFT multiple sequence alignment software version 7: improvements in performance and usability. *Mol Biol Evol*. 2013; **30**(4): 772-780. doi: 10.1093/molbev/mst010
39. Rice P, Longden I, Bleasby A. EMBOSS: the European Molecular Biology Open Software Suite. *Trends Genet*. 2000; **16**(6): 276-277. doi: 10.1016/s0168-9525(00)02024-2
40. Zhang QJ, Gao LZ. Rapid and Recent Evolution of LTR Retrotransposons Drives Rice Genome Evolution During the Speciation of AA-Genome *Oryza* Species. *G3 (Bethesda)*. 2017; **7**(6): 1875-1885. doi: 10.1534/g3.116.037572
41. Baucom RS, Estill JC, Leebens-Mack J *et al.* Natural selection on gene function drives the evolution of LTR retrotransposon families in the rice genome. *Genome Res*. 2009; **19**(2): 243-254. doi: 10.1101/gr.083360.108
42. Vitte C, Panaud O, Quesneville H. LTR retrotransposons in rice (*Oryza sativa, L.*): recent burst amplifications followed by rapid DNA loss. *BMC Genomics*. 2007; **8**: 218. doi: 10.1186/1471-2164-8-218
43. Danecek P, Auton A, Abecasis G *et al.* The variant call format and VCFtools. *Bioinformatics*. 2011; **27**(15): 2156-2158. doi: 10.1093/bioinformatics/btr330
44. Chen ZL, Meng JM, Cao Y *et al.* A high-speed search engine pLink 2 with systematic evaluation for proteome-scale identification of cross-linked peptides. *Nat Commun*. 2019; **10**(1): 3404. doi: 10.1038/s41467-019-11337-z
45. Shabalin AA. Matrix eQTL: ultra fast eQTL analysis via large matrix operations. *Bioinformatics*. 2012; **28**(10): 1353-1358. doi: 10.1093/bioinformatics/bts163

46. Yang Y, Zhang C, Zhu D *et al.* Identifying candidate genes and patterns of heat-stress response in rice using a genome-wide association study and transcriptome analyses. *The Crop Journal*. 2022; **10**(6): 1633-1643. doi: https://doi.org/10.1016/j.cj.2022.02.011
47. Zhang Z, Li J, Pan Y *et al.* Natural variation in *CTB4a* enhances rice adaptation to cold habitats. *Nat Commun*. 2017; **8**: 14788. doi: 10.1038/ncomms14788
48. Zhou X, Stephens M. Genome-wide efficient mixed-model analysis for association studies. *Nat Genet*. 2012; **44**(7): 821-824. doi: 10.1038/ng.2310
49. Dong SS, He WM, Ji JJ *et al.* LDBlockShow: a fast and convenient tool for visualizing linkage disequilibrium and haplotype blocks based on variant call format files. *Brief Bioinform*. 2021; **22**(4). doi: 10.1093/bib/bbaa227
50. Spence JP, Song YS. Inference and analysis of population-specific fine-scale recombination maps across 26 diverse human populations. *Sci Adv*. 2019; **5**(10): eaaw9206. doi: 10.1126/sciadv.aaw9206
51. Kamm JA, Spence JP, Chan J *et al.* Two-Locus Likelihoods Under Variable Population Size and Fine-Scale Recombination Rate Estimation. *Genetics*. 2016; **203**(3): 1381-1399. doi: 10.1534/genetics.115.184820
52. Ashburner M, Ball CA, Blake JA *et al.* Gene ontology: tool for the unification of biology. The Gene Ontology Consortium. *Nat Genet*. 2000; **25**(1): 25-29. doi: 10.1038/75556
53. Cantalapiedra CP, Hernández-Plaza A, Letunic I *et al.* eggNOG-mapper v2: Functional Annotation, Orthology Assignments, and Domain Prediction at the Metagenomic Scale. *Mol Biol Evol*. 2021; **38**(12): 5825-5829. doi: 10.1093/molbev/msab293
54. Chen C, Chen H, Zhang Y *et al.* TBtools: An Integrative Toolkit Developed for Interactive Analyses of Big Biological Data. *Molecular Plant*. 2020; **13**(8): 1194-1202. doi: https://doi.org/10.1016/j.molp.2020.06.009
